# Cost of Deconstruction Depots for Diversified, Waste-Based Lignocellulosic Sugars Using Distillable Solvents

**DOI:** 10.1021/acssuschemeng.5c05029

**Published:** 2025-08-05

**Authors:** Nawa Raj Baral, Xueli Chen, Joseph M. Palasz, Ramkrishna Singh, Anagha Krishnamoorthy, Venkataramana R. Pidatala, Tyrell S. A. Lewis, Chang Dou, Ling Ding, Hemant Choudhary, Ning Sun, Blake A. Simmons, Corinne D. Scown

**Affiliations:** a Biological Systems and Engineering Division, 1666 Lawrence Berkeley National Laboratory, Berkeley, California 94720, United States; b Joint BioEnergy Institute, Lawrence Berkeley National Laboratory, Emeryville, California 94608, United States; c Advanced Biofuels and Bioproducts Process Development Unit, 1666 Lawrence Berkeley National Laboratory, Emeryville, California 94608, United States; d Energy and Environment Science & Technology, 17212 Idaho National Laboratory, Idaho Falls, Idaho 83415, United States; e Bioresource and Environmental Security, 111651 Sandia National Laboratories, Livermore, California 87185, United States; f Energy Analysis and Environmental Impacts Division, 1666 Lawrence Berkeley National Laboratory, Berkeley, California 94720, United States; g Energy and Biosciences Institute, University of California, Berkeley, Berkeley, California 94720, United States

**Keywords:** herbaceous biomass, woody biomass, biomass pretreatment, enzymatic hydrolysis, distillable solvent recovery, lignocellulosic sugar, sugar recovery

## Abstract

Transitioning to a bioeconomy that makes use of low-emission and waste feedstocks requires greater flexibility to accommodate seasonal variations and mitigate long-term storage challenges, such as material loss and fire risk. To achieve this goal, biomass deconstruction technologies must efficiently handle diverse feedstocks. Here, we assess the cost of using butylamine—a distillable solvent—to deconstruct 22 different biomass feedstocks: 7 herbaceous, 9 woody, 4 food processing residues, and 2 blends. Lignocellulosic sugar production costs, based on current empirical data, range from $1.3 to 6.1/kg, suggesting that substantial improvements are required to compete with conventional sugars. The high solvent loading (850 g/kg of whole slurry) is a process bottleneck. Lowering the solvent loading to 59 g/kg of whole slurry, demonstrated in an L-scale reactor using poplar biomass, reduces the minimum sugar selling price by 33%. Solvent loading and recovery, solid loading, sugar yield, enzyme use, and delivered biomass cost all play key roles in reaching sugar production costs of $0.45–0.79/kg. Strategic feedstock blending to maximize carbohydrate content, process optimization to improve conversion efficiency, and the selection of low-cost feedstocks are important to advancing feedstock-flexible biorefineries.

## Graphical abstract

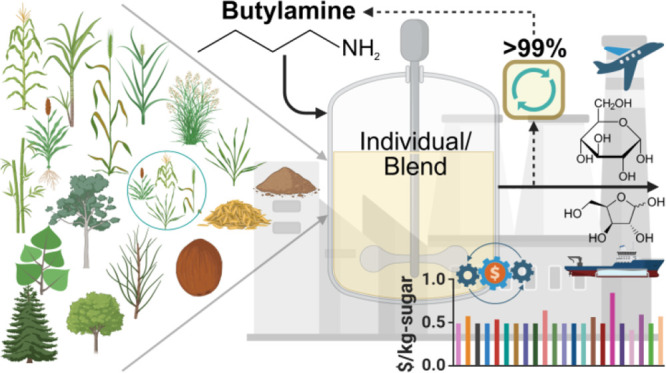

## Introduction

Biomass feedstock availability and type vary across time and region.^1^ This poses substantial challenges to the uninterrupted operation of biobased synthetic fuel and chemical production industries that rely on a single biomass feedstock—particularly during periods of limited availability, price spikes, supply inconsistencies, and quality or quantity losses associated with year-round storage.^2,3^ If facilities can be designed such that they are capable of utilizing a wide range of biomass feedstocks, they will be more flexible and easier to deploy, as they can operate using feedstock blends, sequential single feedstocks, or both.^3,4^ Thriving feedstock-flexible biobased industries require a robust pretreatment method to release fermentable sugars—primarily glucose and xylose—from lignocellulosic biomass, a critical first step in the production of desired synthetic fuels and chemicals. Recently explored butylamine-based pretreatment studies^5,6^ have demonstrated promising sugar yields across diverse biomass types, including agricultural residues, woody biomass, dedicated energy crops, and their blends. This study presents a rigorous technoeconomic analysis (TEA) of a butylamine-based biomass deconstruction process, evaluating 20 individual biomass feedstocks and 2 blends.

In addition to its effectiveness in deconstructing diverse biomass feedstocks, another key advantage of the butylamine-based process is its high solvent recovery rate of over 99%,^5^ enabled by its low boiling point of 78 °C, which allows almost all the solvent to be easily reusable. These advantages set it apart from some of the other leading pretreatment methods—primarily chemical approaches^7–10^ —including alkalis, dilute acids, ionic liquids (ILs), deep eutectic solvents (DESs), and other amine-based distillable solvents^11^ such as ethanolamine. Additionally, scientific efforts to advance promising pretreatment methods and explore complementary strategies for efficient biomass deconstruction remain an active area of research.^7,12,13^ Prior studies^4,14–18^ evaluating the economic impact of leading pretreatment methods have provided valuable insights, but both experimental work and TEAs have largely focused on a few common biomass feedstocks, such as corn stover, miscanthus, switchgrass, biomass sorghum, pine, and poplar. This study compares the economics of a biomass deconstruction process across 22 diverse feedstocks using experimentally determined operating parameters, sugar yields, solvent recovery rates, and consistent process modeling conditions.

We developed a field-to-sugar depot model that produces fermentable sugars—primarily glucose and xylose—while recycling wastewater through anaerobic followed by aerobic treatment and burning lignin onsite for heat and power. These decentralized sugar depots can be located near biomass sources and supply standardized sugar intermediates to the central biorefinery. A biorefinery producing synthetic fuels or chemicals could source sugars from multiple depots, each processing either the same feedstock or different feedstocks. This study investigates whether butylamine-based biomass deconstruction can produce cost-effective lignocellulosic sugars from 22 different biomass types. We also explore the practical limits of a fully optimized butylamine-based process, identify process bottlenecks, highlight improvement opportunities, and identify the critical performance metrics required to meet the cost targets. These insights—gained through conducting an early-stage TEA, even before full process optimization—support informed process improvement, more effective allocation of time and resources to achieve performance targets, strategic feedstock selection, and the potential incorporation of additional biomass types, thereby laying the foundation for feedstock-flexible biobased industries.

## Methods

### Modeling Overview

The lignocellulosic sugar depot model developed in this study integrates an upstream biomass feedstock supply model (built in Microsoft Excel) with a downstream sugar production model created by using SuperPro Designer V13 (Figure 1). The sugar depot is assumed to process 400 bone-dry metric tons (bdt) of biomass per day and is situated near the biomass source, within a 60 km supply radius. The economically optimal depot size is determined through an economy of scale analysis presented Figure S1. The supply distance of 60 km is estimated based on biomass yield and the availability of common feedstocks, including corn stover, miscanthus, switchgrass, and biomass sorghum, and insights from similar studies on woody biomass.^19,20^ Prior studies provide detailed descriptions of the biomass supply chain and sugar production processes.^4,14^ The following sections highlight the key modifications made in this study, and Table 1 summarizes the major data inputs used to develop the process model.

**Figure 1:**
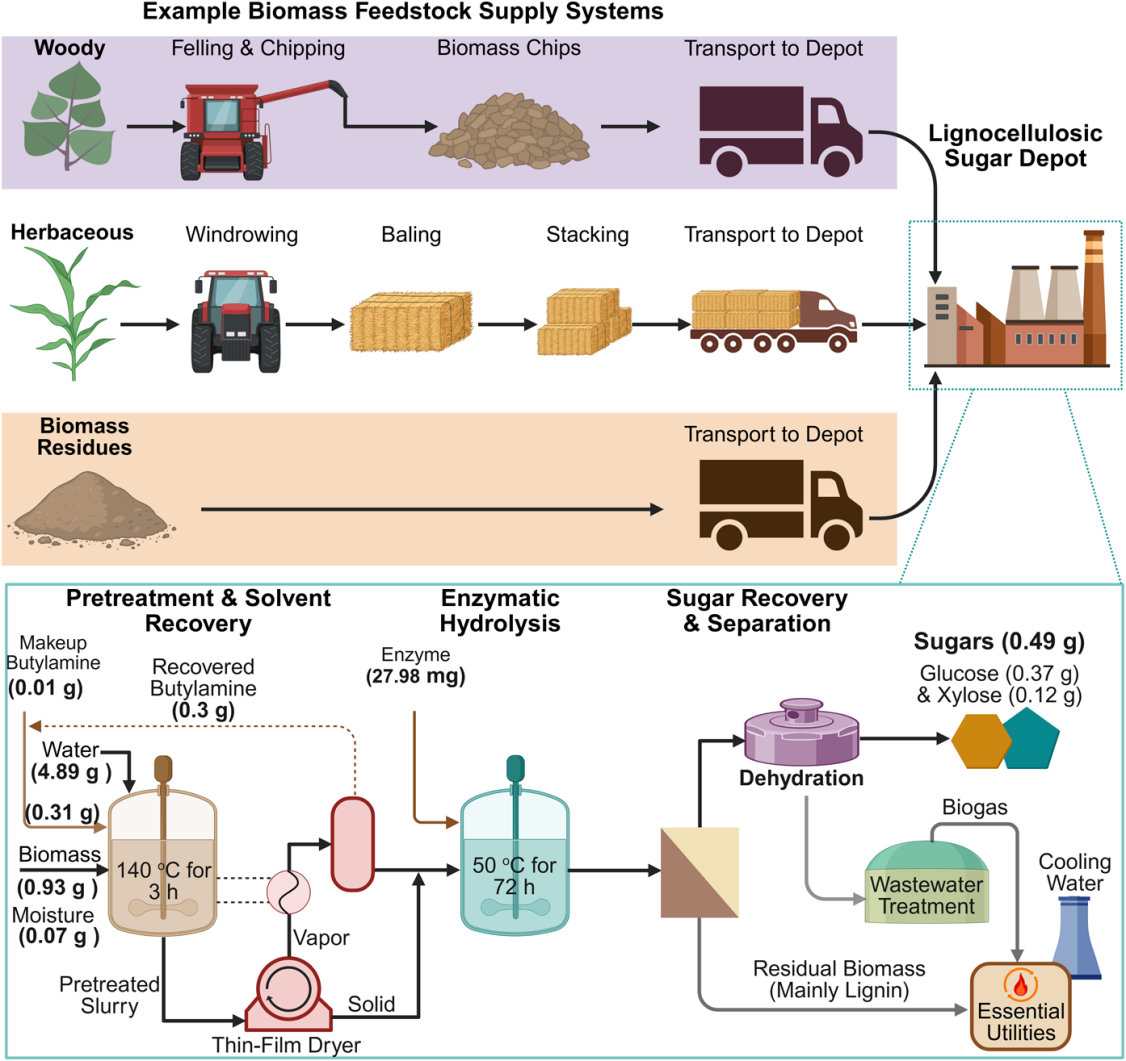
Overview of a biomass-feedstock-flexible lignocellulosic sugar production depot. Values in parentheses represent experimental data and results with poplar biomass using 5 wt % butylamine loading and 15 wt % bone-dry biomass loading, based on the total mass of slurry, as a representative example. A detailed process model for biomass preprocessing, pretreatment, and solvent recovery—key areas of change in this study—has been provided in the Supporting Information (Figures S2 and S3).

**Table 1:** Major Inputs Used to Develop the Field-to-Sugar Process Model in This Study

parameter	unit	current state of technology	optimal future case
sugar depot size^a^	bone-dry metric ton (bdt) biomass intake/day	400	400
feedstock cost	$/bdt	23–250^c^	22–88.2 ^20^ ^c^
**biomass composition**			
glucan	wt %	24.8–45^d^	24.8–45^d^
xlan	wt %	11.5–25.9^d^	11.5–25.9^d^
lignin	wt %	23.7–43.5^d^	23.7–43.5^d^
**biomass pretreatment**			
solid loading rate^5^	wt %	15	30
butylamine loading rate^5^	wt %	85	5^f^
butylamine cost^29^	$/kg	1.5	1
residence time^5^	h	3	1.5
temperature^5^	°C	140	140
**solvent recovery**			
butylamine recovery rate	%	93–99.2^e^	99
**enzymatic hydrolysis**			
enzyme loading rate^5^	mg/g-initial biomass	30	5
solid loading rate^5^	wt %	15	25
cellulose to glucose^5^	%	40.6–93.3^e^	95
xylan to xylose^5^	%	24.8–82.4^e^	90
hydrolysis time^5^	h	72	48
enzyme price^23^	$/kg-protein	5	4
temperature^5^	°C	50	50
**sugar recovery and concentration**			
sugar recovery^b^	%	95	98
**wastewater treatment** ^14,26^			
organic matter to biogas	wt %	86	86
nutrient for AD	$/kg	0.7	0.7
nutrient loading rate	wt %	0.05	0.05
**onsite energy and utilities** ^14,26^			
boiler chemicals cost	$/kg	5	5
water cost	$/kg	0.00022	0.00022
clean-in-place chemical	$/kg	0.53	0.53

a Determined in this study (Figure S1).

b Assumed for analysis in this study.

c The delivered costs of individual biomass feedstocks are documented in Table S1. For the optimal case, the biomass feedstock cost is assumed to be the target cost of $88.2 per metric ton ($80/ton),^20^ unless the actual cost is lower. The structural composition of individual feedstocks is documented in Table S1.

d The structural composition of individual feedstocks is documented in Table S2.

e Both sugar yield and solvent recovery from individual feedstock pretreatments are documented in Table S3.

f Figure S4 presents a representative case demonstrating the comparable effectiveness of butylamine at lower concentrations. Performance data inputs related to biomass composition, pretreatment, solvent recovery, and enzymatic hydrolysis under the current state-of-technology scenario are experimentally validated.

### Delivered Biomass Feedstock Cost and Structural Composition

The delivered costs of most biomass feedstocks, including corn stover, miscanthus, biomass sorghum, switchgrass, wheat straw, pine, and poplar, were based on a prior biomass supply system model developed at the Lawrence Berkeley National Laboratory,^21^ Idaho National Laboratory,^20,22^ and other collaborative studies.^4,19,23^ Additionally, the delivered costs of energy cane, almond stems, walnut stems, and sugar cane bagasse were estimated using farm gate prices reported in the Billion Ton Study,^1^ along with a transportation model developed in this work that assumes a trucking distance of 60 km—consistent with other biomass feedstocks considered (Table S1). The delivered costs of other biomass feedstocks, including hay, bamboo, eucalyptus, hardwood sawdust, oil palm fiber, coconut chips, and rice hulls, were obtained from various sources, as documented in Table S1. In addition to delivered feedstock costs, the structural compositions of the delivered biomass feedstocks from a recent study^5^ are summarized in Table S2.

### Biomass Handling and Preprocessing

In our model, herbaceous biomass, transported in the form of bales, is moved via a conveyor belt to a shredder and subsequently to a hammer mill to reduce it to the desired particle size of 6.35 mm (0.25 in.).^24^ Woody biomass feedstocks except for sawdust, delivered to the sugar depot as chips with an average size of 50.8 mm (2 in.),^20^ also undergo a two-stage milling process^22^ to achieve the same particle size of 6.35 mm. The hardwood sawdust samples we received primarily consisted of fine particles; therefore, our model assumes that milling is required for 5% of the total material, assuming that 5% of the sawdust contains particles larger than the desired size.^25^ Two-stage milling is also applied to coconut chips and oil palm fibers, as the initial biomass samples were typically in the size range of 1–2 in. for coconut chips and 4–6 in. for oil palm fibers.^5^ Other biomass feedstocks, such as sugar cane bagasse and rice husk, which were already at or below the desired particle size, are excluded from further milling in our model.^5^ The milled biomass is temporarily stored before being sent to the biomass deconstruction unit. Assumptions and data sources for biomass preprocessing are consistent with prior studies.^24,26^

### Biomass Pretreatment

Milled biomass is mixed with butylamine to achieve a slurry containing 15 wt % biomass and 85 wt % butylamine,^5^ based on total mass of slurry, before being fed into the reactor. In the scenario where 85 wt % butylamine is used, the solid refers to wet biomass (i.e., biomass with moisture). In cases in which additional water is added to adjust the solid loading, the solid refers to bone-dry biomass. Experimentally measured moisture contents of the selected biomass feedstocks are summarized in Table S2. To better understand performance at lower solvent loadings, our prior study^6^ experimentally evaluated the deconstruction efficiency at varying butylamine-to-water ratios, including a butylamine loading as low as 5 wt % based on total mass of slurry using poplar as a representative biomass feedstock. This prior study^6^ serves as the basis for estimating sugar production costs in the optimal future case and for defining threshold values for key process parameters. Experimental results at different butylamine-to-water mass ratios^6^ are presented in the Supporting Information (Figure S4). The pretreatment reactor is designed for a pressure 1.5 times the saturated pressure of the butylamine–water mixture. Pretreatment is carried out at 140 °C for 3 h.^5,6^ The impact of the pretreatment time was assessed through sensitivity analysis. Following pretreatment, the entire slurry is transferred to the solvent recovery unit.

### Solvent Recovery

Butylamine is recovered using a thin-film dryer, which evaporates volatile chemicals and water while simultaneously conveying solid biomass. This type of system is capable of handling solid loadings up to 60%.^27^ The recovered butylamine is purified through distillation and then recycled back to the pretreatment unit. Experimental recovery rates are documented in Table S3, where 93–99% recovery of butylamine was achieved without additional water washing of the pretreated biomass.^5^ Solvent removal in the experiment was performed via vacuum drying at 40 °C, while personal communication with an industry leader reported over 99% recovery of a distillable solvent using a thin-film drying system. Experimental data were used for baseline analysis, and a 99% recovery rate was considered for determining the optimal selling price of the sugar. As an early-stage technology and based on communication with industry leaders, the thin-film drying system is estimated to cost approximately $4–6 million, with a typical throughput of about 12 t of whole slurry per hour. We applied the learning curve method to estimate costs for an *n*th plant scenario (eq S1), determining the cost of the 1000th unit at $649,200 for a throughput of 12 t/h. Additionally, electricity consumption is assumed to remain constant at 0.063 kWh/kg of processed slurry, as observed in the first unit. Electricity is required to rotate the drum, and steam is used as the heat source to evaporate the solvent. This type of system typically retains 1–15% moisture;^27^ in our model, we assumed 10% moisture (water) retention in the biomass. Other equipment sizing and scaling methods are consistent with those used in previous studies.^14,26^ Alternative recovery methods—such as flash evaporation followed by drying the solid fraction using vacuum drying, sludge drying, drum drying, or rotary drying—could be viable options for butylamine recovery given its relatively low boiling point (78 °C). A comparative assessment of these techniques at an industrial scale would be valuable; however, evaluating their large-scale performance—requiring data on recovery yields and the quality of the dried pretreated material—warrants a separate study with more in-depth analysis and discussion.

### Enzymatic Hydrolysis

Pretreated biomass is mixed with enzymes at a loading rate of 30 mg protein per gram of initial biomass (a 9:1 (v/v) cocktail of CTec3/Htec3), along with water to maintain a solid loading of 15 wt %, and then fed into the enzymatic hydrolysis reactor.^5^ The pH is adjusted to 5 using sulfuric acid. Hydrolysis is carried out at 50 °C for 72 h.^5^ Sugar release during the hydrolysis process, based on our recent study,^5^ is presented in the Table S3. The enzyme loading used in the current state of technology scenario is higher than that used in other processes, such as dilute sulfuric acid pretreatment—for example, 20 mg protein per gram of glucan.^26^ We addressed this by performing a sensitivity analysis. One of the objectives of this study was to determine the threshold enzyme loading required to produce sugar at a cost comparable to those of other common pretreatment methods reported in the literature.

### Sugar Recovery and Concentration

The solid–liquid mixture is separated using a vacuum press filter, which involves filtration, cake washing, and drying steps. The solid fraction is sent to the onsite energy generation unit, while the liquid fraction is directed to the sugar concentration unit. Dilute sugar is concentrated using a mechanical vapor compression system, consistent with prior work.^14^ For the baseline scenario, a 95% recovery rate is assumed. After sugar recovery, the wastewater is routed to a wastewater treatment unit.

### Wastewater Treatment, Onsite Energy, and Utilities

Wastewater is treated using a conventional approach consisting of anaerobic digestion followed by aerobic treatment, consistent with prior studies.^26,28^ Residual biomass, primarily lignin and biogas generated from waste sugar during anaerobic digestion, is burned in a boiler to generate process steam. The remaining process steam is sent to a steam turbine to generate electricity. If on-site electricity generation is insufficient, additional power is sourced from the grid, with any excess electricity sold back to the grid. The utility unit includes the makeup process water pumping, storage, and delivery system as well as the cooling tower, chilled water system, and clean-in-place supply unit, in line with prior studies.

### Determination of the Minimum Selling Price

The minimum selling price of sugar was determined by conducting a discounted cash flow rate of return (DCFROR) analysis^14^ using capital and operating costs and considering recovered sugar on a bone-dry basis (excluding moisture). The capital and operating costs were determined after rigorous material and energy balance analyses. Equipment costs from previous studies were updated based on revised sizing and their scaling exponents.^14,26^ These costs were then converted to 2022 United States dollars using the Chemical Engineering Plant Cost Index. The total capital investment was estimated using cost factors aligned with those reported in earlier studies. Annual operating costs accounted for biomass feedstock, process chemicals, waste disposal, utilities, and fixed expenses such as labor, maintenance, property taxes, and insurance. This study considered an internal rate of return (IRR) of 10% after taxes, a sugar depot lifetime of 30 years, 7920 annual operating hours (330 days/year at 24 h/day), and an income tax rate of 21%.^14,23^ Other economic parameters used in the DCFROR analysis were consistent with those reported in previous technoeconomic studies.^14,26^

## Results and Discussion

### Minimum Sugar Selling Price

Butylamine can effectively deconstruct diverse biomass feedstocks, including herbaceous and woody biomass, as well as their residues and blends.^5,6^ The minimum selling price of lignocellulosic sugar, as presented in Figure 2A, provides evidence of this effectiveness, although the cost of butylamine remains one of the major contributors to sugar production cost at present, as shown in Figure 2B. Results show that the minimum selling price of sugar at the current technological stage ranges from $1.3 to 6.1/kg of mixed sugars. Figure 2A also shows the equivalent prices per kilogram of glucose if a buyer only values the hexose sugars (e.g., because a microbial host cannot co-ferment glucose and xylose). Eucalyptus and coconut chips result in higher sugar selling prices, primarily due to lower biomass-to-sugar conversion yields. This is attributed not only to lower glucose and xylose yields but also to lower cellulose and hemicellulose contents in these feedstocks. With a few exceptions, the minimum selling price of sugar from several other biomass feedstocks falls within the range of $1.3 to 3.9/kg, with an average of $2.6 ± 0.6/kg. This average minimum selling price of sugar increases to $3.6 ± 0.8/kg under a conservative assumption, in which the buyer values only the hexose sugars (Figure 2A). The cost of lignocellulosic sugar production from other common pretreatment methods has been reported to range from $0.31 to 1.41/kg, with an average of $0.60 ± $0.30/kg across studies.^4,14,16–18^ This underscores the need to improve butylamine-based processes to enable the cost-effective production of lignocellulosic sugars. Nonetheless, continued research and development are essential to further reduce sugar production costs from diverse biomass feedstocks and expand the portfolio of economically viable fuels and chemicals.

**Figure 2:**
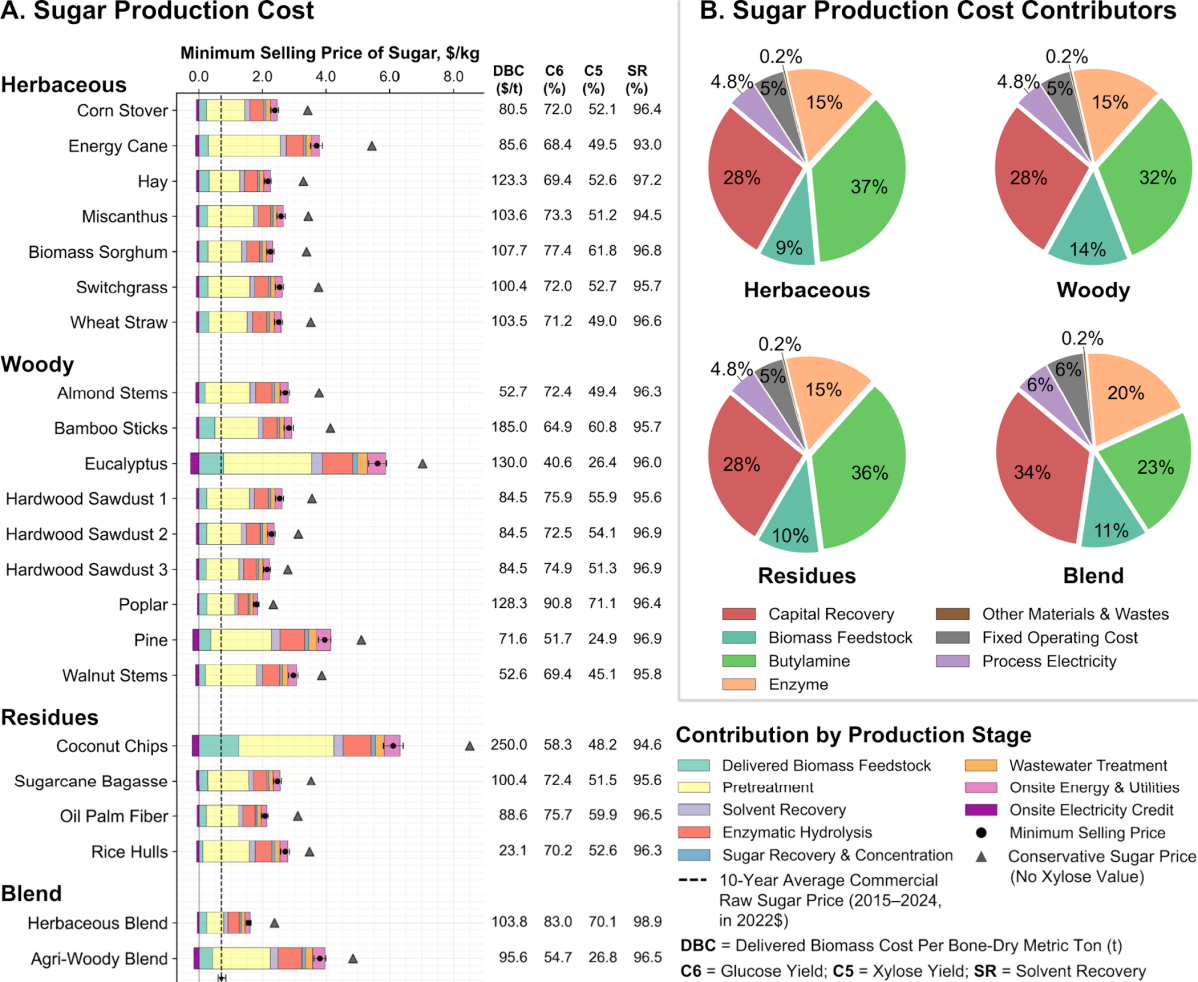
Minimum lignocellulosic sugar selling price (A) across various biomass feedstocks and (B) major contributors to sugar production cost, both based on demonstrated experimental conversion efficiencies (Table 1and Table S3). All experimental results used in this analysis, including sugar yield and solvent recovery, were consistently generated using 15 mL pressure tube setups.^5^ Sugar yields and solvent recovery are listed as a percentage of the theoretical maximum. The error bar associated with the vertical dashed line (- - - -) represents the range of wholesale prices for commercial raw sugar in the United States from 2015 to 2024, referenced to the analysis year 2022 ($0.60 to 0.84/kg).^30^

Among the selected biomass feedstocks, a herbaceous blend—comprising equal amounts of corn stover, biomass sorghum, hay, and wheat straw—resulted in one of the lowest sugar selling prices. Unlike the other feedstocks, which were deconstructed in 15 mL pressure tubes, the herbaceous blend was processed at a 15 mL-to-liter scale because proper mixing in small tubes is nearly impossible. Our recent study^5^ found that deconstructing the herbaceous blend at the 1 L scale results in higher glucose and xylose yields and improved solvent recovery, which together reduce the sugar production cost (Figure 3A). The primary reason for the increased sugar yield is better mixing, which enhances heat and mass transfer during pretreatment and enzymatic hydrolysis. This indicates that even under current operating conditions, simply increasing the scale or, more importantly, improving mixing between biomass and solvent can substantially enhance the efficiency of biomass-to-sugar conversion. This strategy is applicable for reducing sugar production costs from other biomass feedstocks as well.

**Figure 3:**
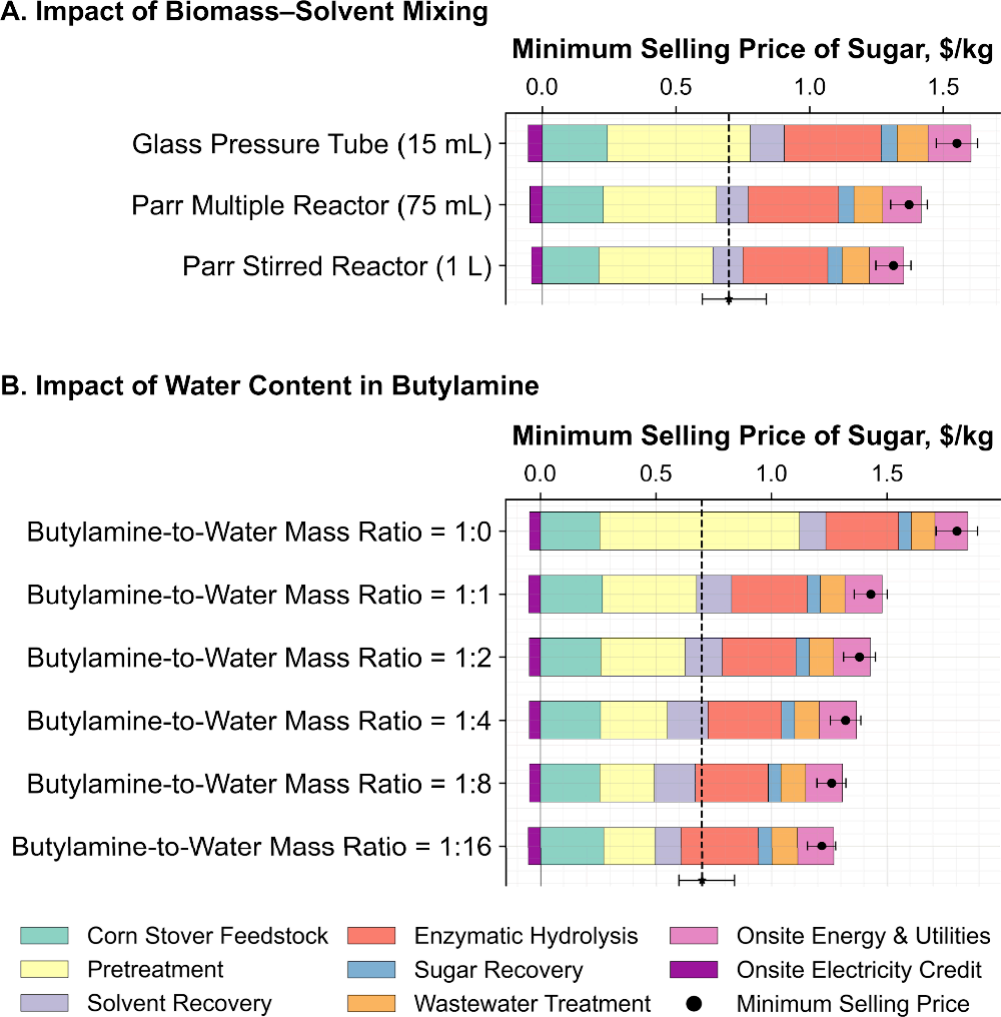
Impact of (A) improved biomass–solvent mixing and (B) water content in butylamine on the minimum selling price of lignocellulosic sugar. Results are based on experimental data from our recent studies. Sugar yields and solvent recovery are listed as a percentage of the theoretical maximum. The vertical dashed line (− – – – ) represents the 10 year average (2015–2024) wholesale price of commercial raw sugar in the United States ($0.69/kg), adjusted to 2022 dollars. The associated error bar indicates a price range of $0.60 to 0.84/kg.^30^

Pretreatment is a major contributor to the total sugar production cost because the amount of makeup butylamine required depends on the butylamine recovery rate. Experimentally obtained butylamine recovery rates vary across feedstocks, ranging from 93 to 99%, as summarized in Table S3. We find that butylamine alone accounts for an average of 32–37% of the total cost—except in the case of the herbaceous blend, where its contribution is reduced to 13%. This reduction is primarily due to about 99% recovery of the solvent. These findings suggest that even a small increase in solvent recovery can substantially lower its cost contribution, which is critical for reducing the minimum selling price of lignocellulosic sugar, especially at the high solvent loading of 85 wt % (based on the whole slurry) used in this work.

To further investigate the impact of solvent loading, we analyzed the effect of low butylamine concentrations (Figure 3B) using recent experimental data with poplar biomass^6^ in which the butylamine-to-water mass ratio was varied. The minimum selling price of sugar was found to decrease with increasing water content or decreasing butylamine loading (Figure 3B). Our recent experimental results show that reducing butylamine loading from 85 to 5 wt % based on the total slurry mass—a 14-fold reduction—led to a decrease in glucose yield from approximately 91 to 84%, while the xylose yield remained steady at 71%. Despite the reduction in glucose yield, the sugar selling price dropped by 32.2% (Figure 3B), underscoring the importance of minimizing solvent loading to reduce overall production costs. Additionally, a lower solvent loading offers three key advantages: it lowers costs, as water is substantially cheaper than organic amines; it reduces the flammability risk associated with organic solvents; and it diminishes the chemical hazards posed by pure butylamine solutions. More importantly, the effectiveness of a diluted butylamine opens the door to further experimentation, as it can serve as a representative compound from the broader family of simple alkylamines, many of which may be viable pretreatment solvents. These alkylamines vary in volatility, toxicity, and cost, presenting different trade-offs.

Following pretreatment, enzymatic hydrolysis is a key contributor to the minimum selling price of sugar. In this work, an enzyme loading of 30 mg of protein/g of initial biomass was used to effectively hydrolyze diverse biomass feedstocks, as woody biomass generally requires more enzymes than herbaceous feedstocks. This highlights substantial room for process optimization by fine-tuning pretreatment temperature, duration, and enzyme loading and by identifying correlations among these parameters.

Solvent recovery in this study was performed using a thin-film drying system, which is capital- and energy-intensive but highly efficient, achieving over 99% solvent recovery. Butylamine, which has a moderate boiling point, may be more easily recovered using alternative methods such as flash evaporation, distillation, vacuum recovery, and condensation or a combination of these systems. While a comparative analysis of different recovery approaches would be valuable, it is beyond the scope of this work. Nonetheless, it presents an opportunity to either improve thin-film drying technology or explore more cost-effective alternatives.

While solvent recovery itself contributes relatively little to the total production cost, the major cost drivers in this process are the biomass feedstock, butylamine solvent, enzyme usage, and capital recovery. Together, these components account for approximately 90% of the total sugar production cost. The low solid loading of 15 wt % in pretreatment and hydrolysis substantially increases the equipment size and cost of these unit operations, as well as those of solvent recovery and wastewater treatment, leading to a higher capital cost contribution to the minimum sugar price. Therefore, targeted optimization of solvent loading, enzyme dosing, and solid loading is essential to reducing the selling price of lignocellulosic sugar.

Biomass feedstock, lignin combustion, and wastewater treatment remain consistent contributors to production costs but are not the focus of this study. While feedstock costs are largely operational (excluding preprocessing), wastewater treatment and on-site energy generation are capital-intensive and contribute substantially to capital recovery costs, which in turn influence the overall selling price of lignocellulosic sugar.

### Sensitivity Analysis of Sugar Production Cost

Figure 4 shows the most influential parameters affecting the minimum selling price of sugar under the current state of the art technology. For this early-stage biomass deconstruction process, the variation in biomass feedstock cost is comparatively less influential. Instead, the sugar production cost is primarily driven by pretreatment and deconstruction parameters.

**Figure 4:**
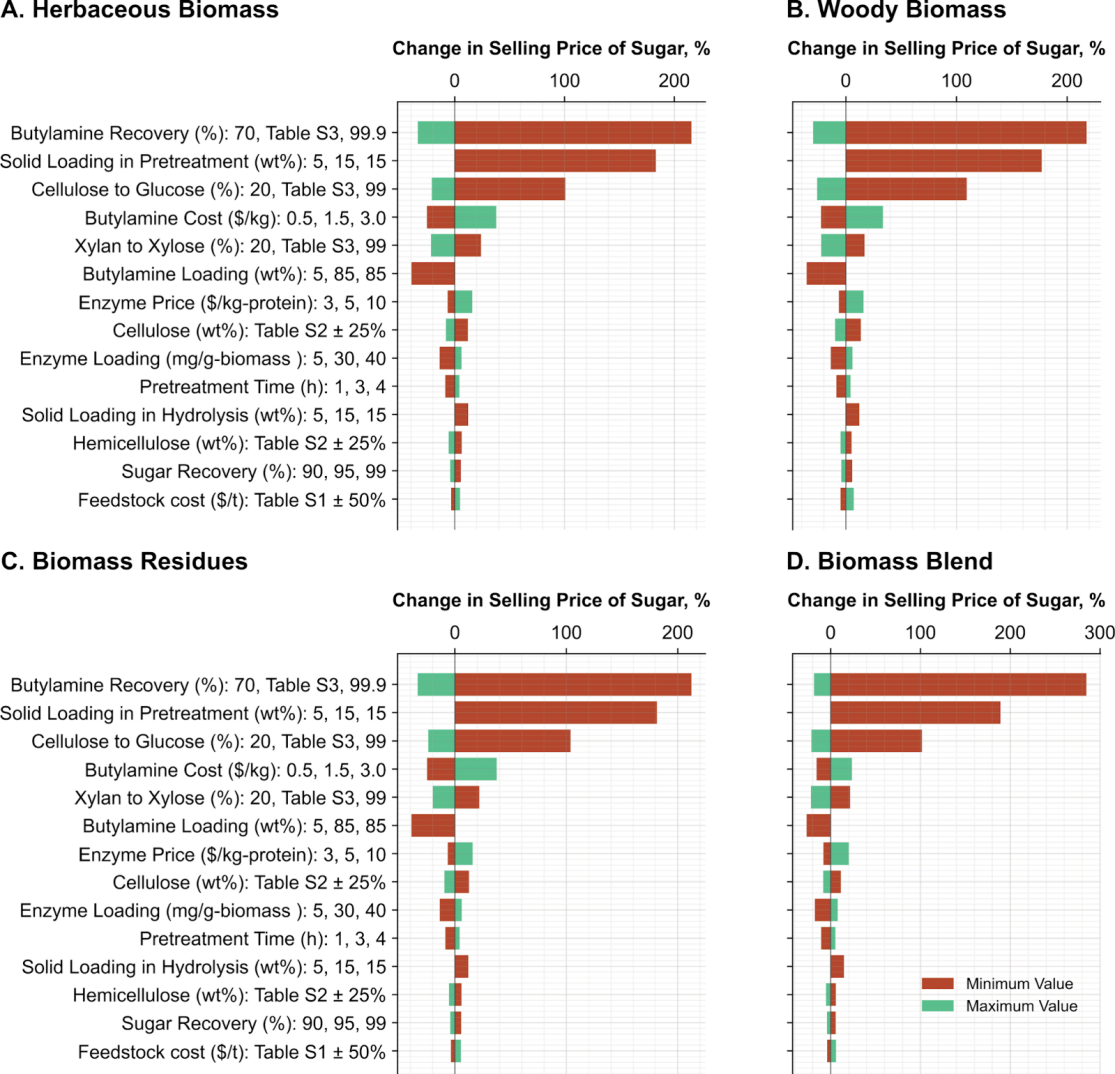
Most sensitive process parameters to lignocellulosic sugar production cost for (A) herbaceous biomass, (B) woody biomass, (C) biomass residues, and (D) biomass blend. The results presented in this figure represent the average impact of each parameter for herbaceous biomass, woody biomass, biomass residues, and biomass blends. To calculate this, sensitivity analyses were performed for each individual feedstock listed under herbaceous, woody, residues, and blend in Figure 1, and the average impact was calculated by combining the results. The individual sensitivity analysis results for each biomass feedstock are very similar to the average presented here and are documented in Figures S5–S26. Sugar yields are reported as a percentage of the theoretical maximum, and solvent recovery rates were measured experimentally and varied across biomass feedstocks (Table S3).

Variability in butylamine loading during pretreatment as well as its recovery efficiency and cost substantially impacts the minimum selling price of sugar. For instance, a 25% decrease in solvent recovery—without changing any other parameters—can increase the sugar cost by more than 200%. This clearly indicates the importance of minimizing the makeup butylamine—by improving recovery efficiency, reducing loading, or both—without compromising biomass deconstruction efficiency—to lower sugar production costs. Additionally, the use of less pure butylamine—such as *n*-butylamine containing *iso*-, *sec*-, and *tert*-butylamines and other organic compounds—can be a cost-effective alternative, as these compounds are easily recoverable due to their lower boiling points compared to those of *n*-butylamine. These mixed or crude solvents are typically cheaper and can substantially reduce the impact of solvent cost even when solvent recovery exceeds 99%. In fact, using low-cost butylamine becomes particularly important when reductions in solvent loading impact biomass deconstruction efficiency.

Solid loading is another critical factor influencing the sugar production cost. Lower solid loadings not only increase the required volume of solvent and the size of solvent recovery systems but also raise the energy demand for solvent recovery. This in turn increases the capital and operating costs of on-site energy and utility stages. Although solid loading during hydrolysis is relatively less influential, it is still important. This is due to the lower energy and cost associated with the mechanical vapor compression system used for sugar concentration compared to the thin-film recovery system adopted in this study for butylamine recovery, which offers high solvent recovery potential. Improving the energy efficiency of the thin-film system or deploying a low-cost, energy-efficient alternative could mitigate the impact of solid loading during pretreatment to the minimum selling price of sugar.

Glucose and xylose yields are clearly important in reducing the minimum selling price of sugar. Altering these yields not only changes the total product volume or sugar produced but also affects the energy requirement for sugar concentration by modifying the sugar concentration in the feed to the recovery unit.

Enzyme loading and its unit cost also substantially influence the overall cost of enzymes required for biomass deconstruction. While low-cost enzymes are challenging to procure, onsite enzyme production as discussed in a prior study^26^ can reduce enzyme costs. This integrated approach benefits from shared downstream infrastructure, such as energy, utilities, and wastewater treatment systems. Further research focused on enhancing the deconstruction efficiency at lower enzyme loadings is essential to reduce the minimum selling price of sugar.

Variations in biomass cellulose and hemicellulose content influence the minimum selling price of sugar primarily by altering the total sugar yield, which, in turn, affects sugar feed concentration and energy requirements for recovery. Pretreatment and hydrolysis times also play important roles, as they can affect the reactor sizing and capital costs. The sugar recovery rate directly impacts the final sugar yield and is, therefore, another key parameter influencing the minimum selling price of sugar.

### Process Optimization Impacts and Optimal Sugar Selling Prices

Optimizing the most influential process parameters has been shown to substantially reduce the cost of sugar production. A stepwise reduction in the minimum sugar selling price is presented in Figure 5A, using poplar as a representative biomass feedstock. Similar stepwise cost reduction results for the herbaceous blend are shown in Figure S27. Butylamine loading was identified as the most influential factor. Figure 5A shows a 32% reduction in the sugar selling price when butylamine loading is decreased from 85 to 5 wt % (based on the total slurry mass) despite our recent experimental results^6^ indicating a glucose yield reduction of over 6% at 5 wt % butylamine loading.

**Figure 5:**
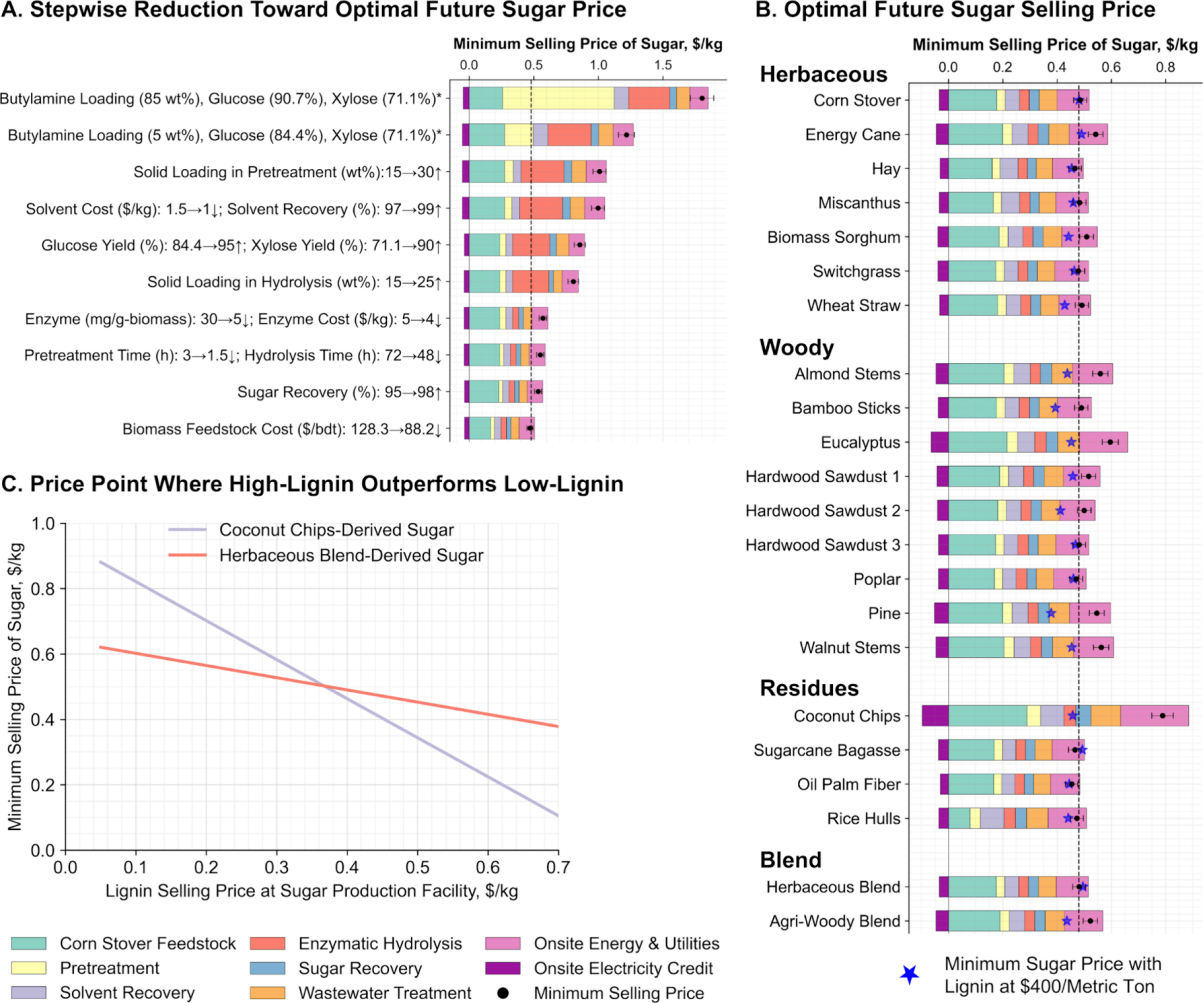
Minimum lignocellulosic sugar selling price in optimal future: (A) stepwise price reduction using poplar as a representative feedstock, (B) optimal prices across diverse biomass feedstocks, and (C) cutoff lignin price at which coconut outperforms herbaceous blend. The vertical dashed lines (- - - -) represent the average reported selling price of corn-stover-derived sugar ($0.48/kg) produced using dilute sulfuric acid pretreatment.^4,31^ *Experimentally measured data.^5,6^

At lower butylamine loadings, one effective approach to reduce the water content in the pretreatment process is to increase solid loading. Excess water not only increases the energy required for solvent recovery but also elevates wastewater treatment costs. Increasing the solid loading from 15 to 30% further reduced the minimum sugar selling price by 14.4%. Additional reductions in production cost were achieved by lowering the solvent loading to 5%, increasing the solvent recovery to 99%, and utilizing low-cost (less pure) butylamine, which collectively contributed to a 4.3% cost reduction.

Further process improvements, such as increasing sugar yield to over 90% of the theoretical maximum and raising solid loading during hydrolysis from 15 to 25%, collectively reduced the sugar production cost by 19%. An even more substantial reduction of 29.4% was observed when enzyme loading was reduced by a factor of 6. The challenge, however, lies in maintaining high sugar yields while operating at lower solvent and enzyme loadings. This may require fine-tuning of other operating parameters, such as pretreatment temperature and duration as well as hydrolysis conditions.

While these parameters are collectively important, they are comparatively less influential than solvent and enzyme loading in determining cost. Nonetheless, improvements in sugar recovery and locating biorefineries close to biomass sources—thereby reducing delivered biomass costs—could further drive down sugar production costs. These improvements collectively result in minimum sugar selling prices below $0.50/kg for 13 out of the 22 selected feedstocks. Additionally, achieving production costs comparable to those of commonly used dilute sulfuric acid (DSA) pretreatment is possible. Applying similar cost-reduction strategies applied for poplar biomass (Figure 5A), several biomass feedstocks can reach cost parity with DSA, except for a few that have inherently lower carbohydrate contents (Figure 5B). Therefore, in addition to feedstock price, the carbohydrate content is a critical factor in determining biomass quality and achieving low-cost sugar production.

In addition to carbohydrates, the lignin content varies across feedstocks, leading to higher costs for on-site energy generation when using high-lignin feedstocks such as coconut chips, eucalyptus, and pine (Figure 5B), which in turn result in a higher selling price for sugar. A notable advantage of the butylamine-based pretreatment method, as demonstrated in recent work,^6^ is that it yields relatively high-quality lignin comparable to lignin obtained from organosolv pretreatment. This suggests a strong potential for selling lignin for high-value applications instead of burning it to generate heat and power.

To assess the impact of the lignin value, we ran a separate scenario that considered lignin recovery and sale. In this scenario, only process steam is generated on-site using natural gas, and all electricity required for the facility is sourced directly from the grid. We found that selling lignin at $400 per metric ton, instead of burning it onsite, results in sugar selling prices below $0.50/kg for all 22 selected feedstocks (Figure 5B). However, for some feedstocks—such as corn stover, the herbaceous blend, and sugar cane bagasse—the sugar selling price remains unchanged or fails to outperform the combustion scenario even at this lignin value (Figure 5B). In contrast, sugar derived from coconut chips, which is typically the most expensive, becomes cheaper than that from the herbaceous blend when the lignin value exceeds $380 per metric ton (Figure 5C). These results underscore that both the lignin content of the biomass and the market value of lignin are important factors in reducing sugar selling prices. Selling lignin rather than combusting it is particularly advantageous for high-lignin feedstocks.

### Performance Thresholds and Future Research Priorities

A unique advantage of butylamine pretreatment is its effectiveness across diverse herbaceous and woody biomass types. To achieve an optimal sugar selling price or reach price parity with lignocellulosic sugars produced from other promising biomass deconstruction methods, we identified threshold values for the most influential process parameters using poplar as a representative feedstock (Figure 6). Our analysis shows that a solid loading below 10 wt % during pretreatment substantially increases sugar production costs even when other parameters are fully optimized. At around 10 wt %, the sugar selling price decreases gradually, but reaching parity with DSA-pretreated lignocellulosic sugar requires a solid loading above 29 wt % (Figure 6A). This is because the solid loading influences the amount of solvent required, which affects both capital and operating costs—not only for pretreatment but also system-wide—including solvent recovery, onsite energy generation, utilities, and wastewater treatment. The amount of solvent determines material and equipment needs, as well as energy consumption. Similarly, solid loadings below 10% during enzymatic hydrolysis lead to substantial increases in the minimum selling price of sugar, with a loading of at least 21% required to achieve cost parity with sugar produced from DSA-pretreated corn stover (Figure 6B).

**Figure 6:**
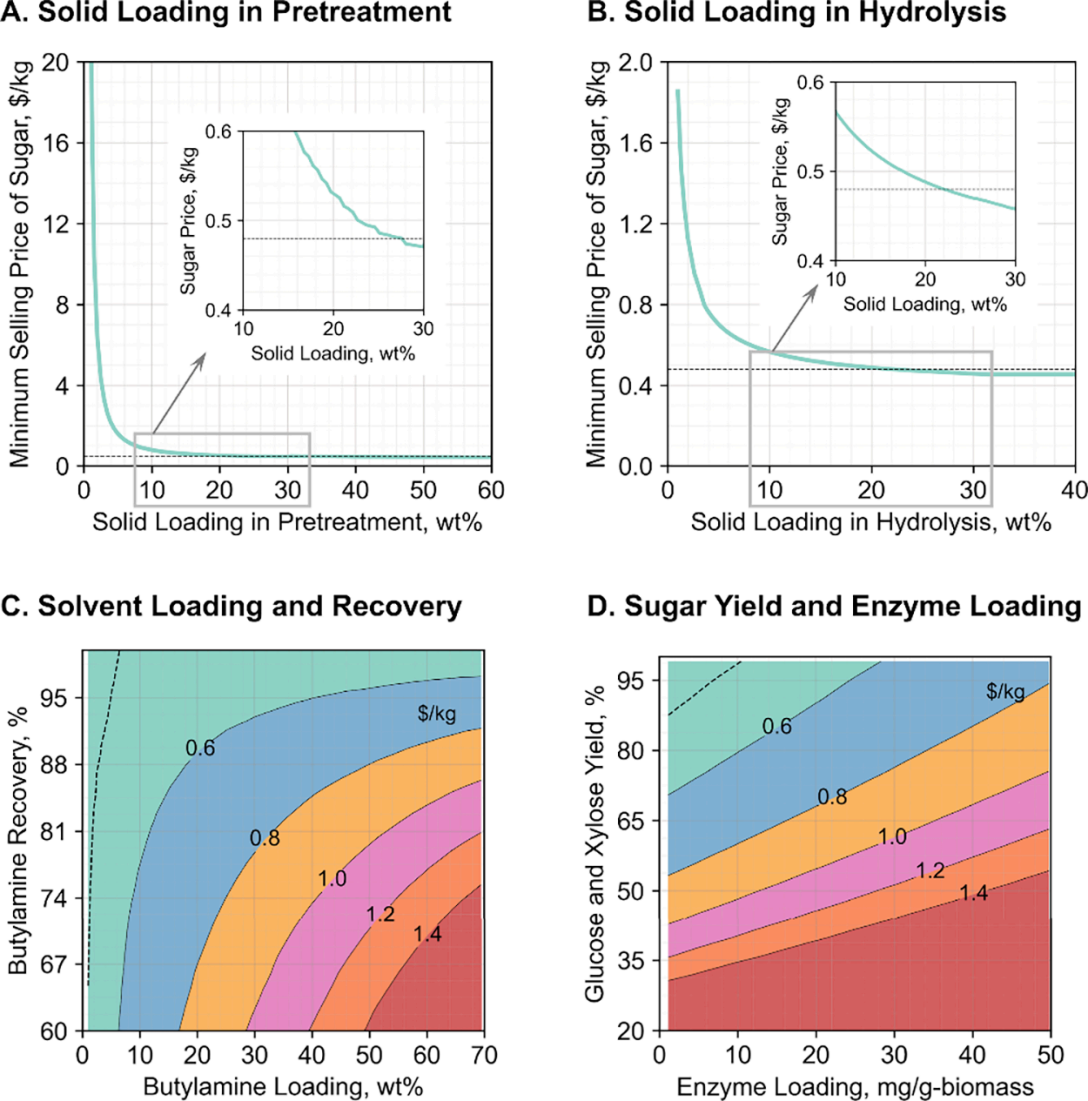
Threshold values of major process parameters associated with biomass deconstruction including (A) solid loading in pretreatment, (B) solid loading in hydrolysis, (C) butylamine loading and recovery, and (D) sugar yield and enzyme loading. These thresholds were determined based on optimal values of other process parameters, as listed in Table 1. The vertical dashed lines (- - - -) represent the average reported selling price of corn-stover-derived sugar ($0.48/kg) produced using dilute sulfuric acid pretreatment.^4,31^

In addition to solid loading, both butylamine loading and recovery must be optimized to reach cost-parity sugar production with the DSA-pretreated corn stover process. The TEA results show that a butylamine concentration as low as 5.9 wt % coupled with a >98% recovery is sufficient (Figure 6C). Experiments with poplar biomass indicate that these targets are realistic. Pushing the loading down to 5 wt % and pushing recovery above 99% also appear attainable and should be priority goals for a wider range of feedstocks. Even incremental changes are valuable, as they buffer against periodic solvent replacement and butylamine price spikes while allowing flexibility to optimize critical parameters, such as enzyme loading and sugar yield.

Enzyme loading and sugar yield remain the strongest cost levers. Reducing the enzyme dosage from the current 30 mg per gram of initial biomass by 5-fold—while still achieving over 90% of the theoretical sugar yield—would dramatically lower operating costs (Figure 6D). Although this benchmark is feasible for some substrates—based on deconstruction results from other solvent-based pretreatments^11^—it may be challenging to achieve across a fully diverse feedstock slate. Fortunately, several less-sensitive knobs can still be turned—pretreatment time and temperature, hydrolysis duration, one-pot hydrolysis without solvent separation, or alternative low-cost, energy-efficient solvent-recovery schemes—allowing the process to achieve the targeted sugar cost while preserving flexibility in the most critical factors.

## Conclusions

Butylamine shows promise as a cost-effective solvent for deconstructing a wide range of biomass feedstocks—including herbaceous, woody, biomass residues, as well as their blends—demonstrating strong potential for feedstock-flexible biorefineries. However, substantial improvements over the current state of technology are needed, particularly in achieving high sugar yields at low enzyme loadings and high solid content. To make the process economically viable and competitive with dilute sulfuric acid pretreatment, key performance thresholds were identified: solid loadings above 29% in pretreatment and 21% in hydrolysis, butylamine loading at or below 5%, butylamine recovery above 99%, enzyme loading of 5 mg of protein/g of initial biomass (nearly 10 mg/g of glucose), and sugar yields exceeding 90% of the theoretical maximum. These improvements result in minimum sugar selling prices below $0.50/kg for 13 out of the 22 selected feedstocks. Additionally, selling lignin at $400 per metric ton instead of burning it onsite results in sugar selling prices below $0.50/kg for all 22 selected feedstocks. Even the most expensive sugar, derived from coconut chips, becomes cheaper than that from the herbaceous blend when the lignin value exceeds $380 per metric ton. Bench-scale results already meet butylamine loading and recovery targets, and further research and development are essential to achieving all thresholds collectively and to validate lignin quality for commercial viability.

## Supplementary Material



## Notes

Delivered biomass feedstock costs; structural composition and moisture content of the feedstocks; glucose and xylose yields and solvent recovery rates from each feedstock experiment; sugar selling prices as a function of depot size; learning curve equation to calculate *n*th equipment price; the process flow diagram, streamflow details, as well as equipment sizing and associated costs for biomass preprocessing, pretreatment, and solvent recovery; sugar yields and butylamine recovery rates at varying butylamine-to-water ratios; sensitivity analysis results for 22 individual feedstocks; and a representative stepwise cost reduction for the herbaceous blend (PDF)
