# Supplementary material for: Cost of Deconstruction Depots for Diversified, Waste-Based Lignocellulosic Sugars Using Distillable Solvents
Source: ACS Sustain Chem Eng. 2025 Aug 5;13(32):13100–11. doi: 10.1021/acssuschemeng.5c05029 (PMC12365919; doi:10.1021/acssuschemeng.5c05029)
Supplement: Supplementary file 1 [file sc5c05029_si_001.pdf]

Supporting Information

Cost of Deconstruction Depots for Diversified, Waste-Based Lignocellulosic Sugars Using Distillable Solvents

Nawa Raj Baral[a,b,*], Xueli Chen[a], Joseph M. Palasz[a], Ramkrishna Singh[a,c], Anagha Krishnamoorthy[a], Venkataramana R. Pidatala[a,b], Tyrell S. A. Lewis[a,c], Chang Dou[a,c], Ling Ding[d], Hemant Choudhary[b,e], Ning Sun[a,c], Blake A. Simmons[a,b], Corinne D. Scown[a,b,f,g]

[a]Biological Systems and Engineering Division, Lawrence Berkeley National Laboratory, Berkeley, California 94720, United States

[b]Joint BioEnergy Institute, Lawrence Berkeley National Laboratory, Emeryville, California 94608, United States

[c]Advanced Biofuels and Bioproducts Process Development Unit, Lawrence Berkeley National Laboratory, Emeryville, California 94608, United States

[d]Energy and Environment Science & Technology, Idaho National Laboratory, Idaho Falls, Idaho 83415, United States

[e]Bioresource and Environmental Security, Sandia National Laboratories, Livermore, California 87185, , United States

[f]Energy Analysis and Environmental Impacts Division, Lawrence Berkeley National Laboratory, Berkeley, California 94720, United States

[g]Energy and Biosciences Institute, University of California, Berkeley, Berkeley, California 94720, United States

*Corresponding author, Email: nrbaral@lbl.gov

Supporting Information includes:

Number of pages: 37

Number of figures: 27

Number of tables: 7

S1. Delivered Biomass Feedstock Costs

Table S1. Delivered biomass feedstock cost

Feedstock	Delivered Cost ($/bone-dry metric ton)	Feedstock	Delivered Cost ($/bone-dry metric ton)
Sorghum[1]	107.71	Sugarcane bagasse[ϕ]	100.44
Corn stover [2]	80.52	Almond stems[3]	52.72[δ]
Wheat straw[α]	103.48	Walnut stems[3]	52.64[δ]
Hay[4,5]	123.33	Hardwood sawdust 1 [6]	84.52
Rice hulls[7,8]	23.15	Hardwood sawdust 2 [6]	84.52
Miscanthus [2]	103.55	Hardwood sawdust 3 [6]	84.52
Switchgrass [2]	100.37	Hybrid poplar[9,10]	128.3
Energy cane[Θ]	85.65	Eucalyptus[11]	130[β]
Bamboo sticks[Υ]	185	Pine[12]	71.62
Oil palm fiber [13]	88.57	Herbaceous blend*	103.8
Coconut chips [14]	250	Agri-woody blend*	95.6

[α]The delivered cost of wheat straw was determined using the corn stover supply logistics model, with key parameters adjusted to reflect wheat straw characteristics. These included a biomass yield of 2.08 metric tons per hectare,[3] an assumed 5% of wheat fields surrounding the sugar depot, and nutrient replenishment values gathered from various sources.[15–17]
[ϕ]Sugarcane bagasse prices vary by region, with reported values ranging from $26 to $50 per metric ton.[18,19] Because bagasse is typically burned in boilers, its price is often benchmarked to the cost of the alternative energy source—usually natural gas—needed to deliver the same thermal output. Using a published regression that relates bagasse price to natural gas price, [20] we estimated the equivalent cost of bagasse for the analysis year 2022. Based on an industrial natural gas price of $7.76 per thousand cubic feet in 2022, [21] the calculated bagasse cost at the sugar factory is $91.86 per bone-dry metric ton. Including a transportation cost for a 60 km trucking radius, the total delivered cost to the sugar depot is $100.44 per bone-dry metric ton.
[Υ]The price of bamboo sticks is assumed to be similar to that of commercially available bamboo residues. (https://www.alibaba.com/trade/search?spm=a2700.details.pageModule_fy23_pc_search_bar.keydown__Enter&tab=all&SearchText=bamboo+chips).
[Θ]We considered the farm-gate price of energy cane from the Billion Ton Study [3] and calculated transportation costs assuming a trucking distance of 60 km. For comparison, the targeted delivered cost in a prior study[22] —less than $92.6 per metric ton—is comparable to the cost estimated in this study.

[β]Reported prices range from $110 to $150 per metric ton, with an average of $130 per metric ton.

[δ]We considered the farm-gate price of noncitrus pruning residues from the Billion Ton Study,[3] and calculated transportation costs assuming a trucking distance of 60 km.

*The delivered costs of the herbaceous and agri-woody blends (comprising agricultural residues and woody biomass) were determined based on their weighted average composition. The herbaceous blend consists of equal parts of corn stover, biomass sorghum, wheat straw, and hay, while the agri-woody blend includes equal parts of corn stover, switchgrass, eucalyptus, and pine.

S2. Structural Composition and Moisture Content of Biomass Feedstocks

Table S2. Structural composition of biomass feedstocks (all data from recent experimental work[23])

Feedstock	Glucan (%)		Xylan (%)		Klason Lignin (%)		Acid Soluble Lignin (%)		Ash (%)		Moisture (%)	
	**Mean**	**SD**	**Mean**	**SD**	**Mean**	**SD**	**Mean**	**SD**	**Mean**	**SD**	**Mean**	**SD**
Sorghum	34.8	0.2	21.5	0.3	20.7	0.2	4.5	0	6.49	0.4	7.21	0.55
Corn stover	37.3	0.4	21.8	0.4	18.5	0.1	4.2	0	4.93	0.31	7.25	0.12
Wheat straw	36.9	0.6	21.1	0.1	17.7	0	5.4	0.1	7.7	0.3	7.53	0.07
Hay	39.2	1	25.9	0.8	21.4	0.1	4.3	0.5	3.39	0.13	8.78	0.14
Rice hulls	35.8	0.6	13.3	0	22	0.1	2.9	0	18.13	0.15	6.68	0.04
Miscanthus	43.3	0.1	20.1	0.2	23.5	0.3	3.9	0	2.2	0.1	7.34	0.13
Energy cane	32.3	0.3	20.5	0.2	26.8	0.2	4.4	0	6	0	9.45	-
Bamboo sticks	39.9	0	19.3	0	25.5	0	4.1	0.1	0.59	0.05	6.56	0.18
Oil palm fiber	38.9	0.1	24.2	0	18.8	0	6	0.1	2.97	0.04	6.69	0.04
Coconut chips	24.8	0.1	11.5	0.1	43.5	0.2	3.7	0	0.042	0.002	10.29	-
Sugarcane bagasse	39	0.1	23.2	0.1	24.6	0.3	4.3	0.1	1.1	0.5	7.58	0.17
Almond stems	32.8	0.5	18.5	0.3	27.5	0	4.3	0	1.64	0.09	6.89	0.08
Walnut stems	35.1	0.7	15.9	0.3	26.2	0.2	5.3	0.1	4.5	0.5	7.34	0.13
Hardwood sawdust 1	36.4	0.5	19.4	0.5	26.4	0.2	4.5	0.4	0.6	0.09	7.86	0.06
Hardwood sawdust 2	38.9	0.2	18.9	0.01	25.8	0.4	4.8	0	0.5	0	8.14	0.19
Hardwood sawdust 3	42.1	0.1	18.2	0	24.1	0.1	4.6	0	0.54	0.33	6.67	0.08
Hybrid poplar	45	0.4	16.8	0.1	23.9	0.1	5.1	0	0.7	0.2	6.72	0.18
Eucalyptus	35.2	0.3	13.2	0	36.3	0.3	5	0.1	1.6	0.1	7.77	0.13
Pine	33.1	0.2	19.6	0.2	33	0.4	2.9	0.1	1.3	0	9.18	0.02

SD = Standard deviation.

S3. Glucose and Xylose Yields, and Solvent Recovery Rates from Individual Feedstock Experiments

Table S3. Solvent recovery and sugar yields from different biomass feedstocks (all data from recent experimental work[23])

Feedstock	Solvent Recovery		Glucose Yield (%)		Xylose Yield (%)	
	Mean	Standard Deviation	Mean	Standard Deviation	Mean	Standard Deviation
Sorghum	96.77	0.28	77.44	1.30	61.80	1.28
Corn stover	96.37	0.34	72.01	0.51	52.06	0.86
Wheat straw	96.56	0.59	71.25	0.61	49.00	2.75
Hay	97.20	0.30	69.36	0.32	52.59	0.22
Rice hulls	96.26	0.87	70.21	1.10	52.56	0.47
Miscanthus	94.51	0.27	73.32	0.83	51.22	0.68
Energy cane	93.01	2.39	68.39	0.38	49.55	1.88
Bamboo sticks	95.71	0.58	64.90	1.71	60.83	1.65
Oil palm fiber	96.51	0.02	75.74	2.75	59.93	1.67
Coconut chips	94.64	0.27	68.02	19.03	52.33	18.13
Sugarcane bagasse	95.62	0.32	72.43	0.51	51.55	2.24
Almond stems	96.31	0.59	72.43	2.19	49.43	2.14
Walnut stems	95.78	0.25	69.44	0.24	45.09	0.93
Hardwood sawdust 1	95.64	0.59	75.88	0.58	55.90	0.03
Hardwood sawdust 2	96.85	0.59	72.55	0.92	54.10	0.32
Hardwood sawdust 3	96.85	0.02	74.87	0.64	51.35	0.50
Hybrid poplar	95.34	0.60	69.94	3.80	38.57	0.41
Eucalyptus	95.96	0.60	40.58	1.32	26.38	1.94
Pine	96.86	0.30	51.73	1.59	24.87	2.71
Herbaceous blend*	99.23	0.39	93.28	1.66	82.45	2.98
Agri-woody blend*	96.53	0.31	54.73	0.37	26.77	1.65

*The herbaceous blend consists of equal parts of corn stover, biomass sorghum, wheat straw, and hay, while the agri-woody blend includes equal parts of corn stover, switchgrass, eucalyptus, and pine.

S4. Sugar Selling Prices as a Function of Depot Size

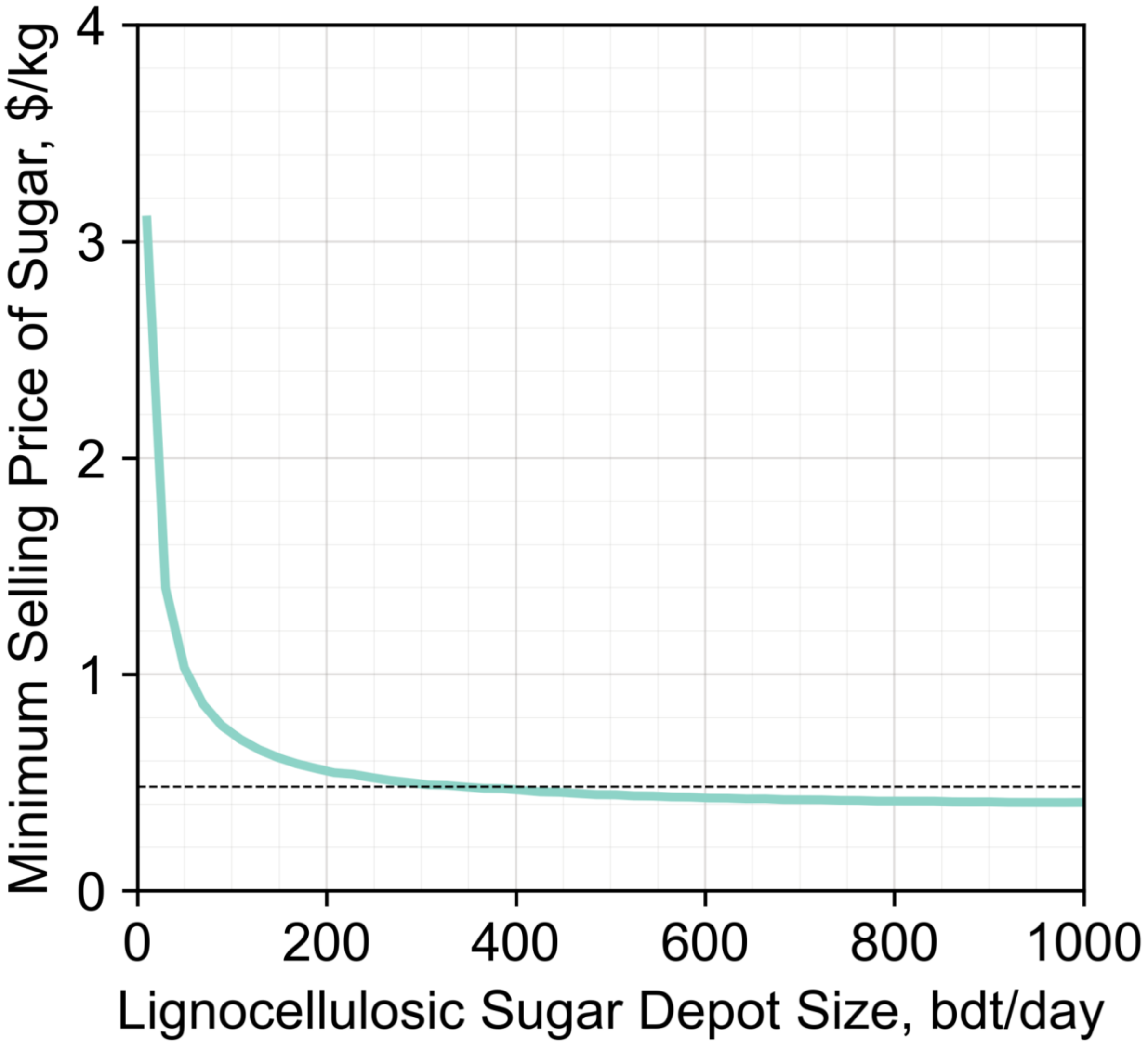

Figure S1. Minimum selling price of sugar as a function of depot capacity. This is a representative example using poplar as the feedstock and the optimal process parameters considered in this study. The horizontal dashed line (- - - -) represents the average reported selling price of corn stover–derived sugar ($0.48/kg) produced using dilute sulfuric acid pretreatment.[2,24]

S6. Butylamine Recovery System Cost

We apply the learning curve method to estimate the capital cost of a thin-film based distillable solvent recovery system for an nth plant scenario. This study considers the cost of the 1000th unit, which is estimated using Equation 1.[26]

$C_n = C_1 \times n^{log_2(b)}$.............**Equation S1**.

C_n = Cost of the nth unit

C_1 = Cost of the first unit ($6,000,000)

n = n^{th} unit number (e.g., 1000 for this study)

b = Learning rate (We assumed an average learning rate of 0.8, which typically falls within the range of 0.7 to 0.9).

Process Flow Diagram and Equipment Cost

This section includes the process flow diagram, stream flow details, equipment sizing, and associated costs for biomass preprocessing, pretreatment, and solvent recovery. These process models are primarily developed based on previous models,[27,28] which provide more detailed information on these stages as well as other stages, including enzymatic hydrolysis, sugar recovery, wastewater treatment, and onsite energy and utilities.

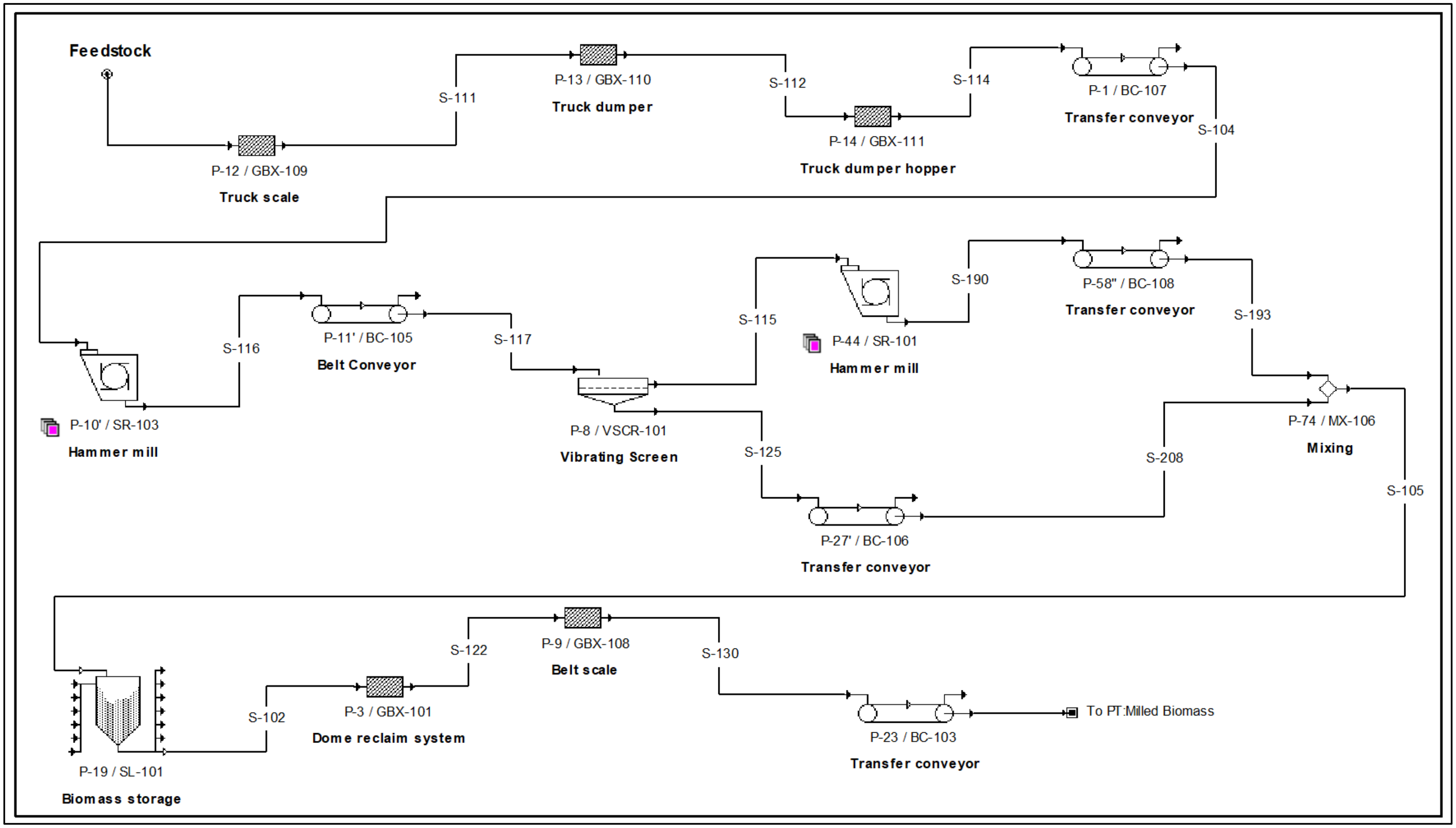

Figure S2. Process flow diagram of the biomass preprocessing stage.

Table S4. Stream flow details associated with the biomass preprocessing stage (see **Figure S2**). These are representative results based on poplar as the biomass feedstock and pretreatment conditions reflecting the current state of technology, as demonstrated in bench-scale experiments (Table 1).

Component	Units	Feedstock	S-111	S-112	S-114	S-104	S-116	S-117	S-115	S-125	S-190	S-193	S-208	S-105	S-102	S-122	S-130	To PT: Milled Biomass
Total Mass Flow	kg/h	17867.4	17867.4	17867.4	17867.4	17867.4	17867.4	17867.4	9438.4	8429.0	9438.4	9438.4	8429.0	17867.4	17867.4	17867.4	17867.4	17867.4
Temperature	°C	25	25	25	25	25	25	25	25	25	25	25	25	25	25	25	25	25
Pressure	bar	1.01	1.01	1.01	1.01	1.01	1.01	1.01	1.01	1.01	1.01	1.01	1.01	1.01	1.01	1.01	1.01	1.01
Acetate	kg/h	706.7	706.7	706.7	706.7	706.7	706.7	706.7	0.0	706.7	0.0	0.0	706.7	706.7	706.7	706.7	706.7	706.7
Acetic-Acid	kg/h	0.0	0.0	0.0	0.0	0.0	0.0	0.0	0.0	0.0	0.0	0.0	0.0	0.0	0.0	0.0	0.0	0.0
Ash	kg/h	116.7	116.7	116.7	116.7	116.7	116.7	116.7	64.2	52.5	64.2	64.2	52.5	116.7	116.7	116.7	116.7	116.7
Butylamine	kg/h	0.0	0.0	0.0	0.0	0.0	0.0	0.0	0.0	0.0	0.0	0.0	0.0	0.0	0.0	0.0	0.0	0.0
Cellulose	kg/h	7500.0	7500.0	7500.0	7500.0	7500.0	7500.0	7500.0	4125.0	3375.0	4125.0	4125.0	3375.0	7500.0	7500.0	7500.0	7500.0	7500.0
Extractives	kg/h	709.8	709.8	709.8	709.8	709.8	709.8	709.8	390.4	319.4	390.4	390.4	319.4	709.8	709.8	709.8	709.8	709.8
Hemicellulose	kg/h	2800.0	2800.0	2800.0	2800.0	2800.0	2800.0	2800.0	1540.0	1260.0	1540.0	1540.0	1260.0	2800.0	2800.0	2800.0	2800.0	2800.0
Lignin	kg/h	4833.3	4833.3	4833.3	4833.3	4833.3	4833.3	4833.3	2658.3	2175.0	2658.3	2658.3	2175.0	4833.3	4833.3	4833.3	4833.3	4833.3
Protein	kg/h	0.2	0.2	0.2	0.2	0.2	0.2	0.2	0.1	0.1	0.1	0.1	0.1	0.2	0.2	0.2	0.2	0.2
Soluble lignin	kg/h	0.0	0.0	0.0	0.0	0.0	0.0	0.0	0.0	0.0	0.0	0.0	0.0	0.0	0.0	0.0	0.0	0.0
Water	kg/h	1200.7	1200.7	1200.7	1200.7	1200.7	1200.7	1200.7	660.4	540.3	660.4	660.4	540.3	1200.7	1200.7	1200.7	1200.7	1200.7

Table S5. Equipment sizing and associated costs for the biomass preprocessing stage (see **Figure S2**). These are representative results based on poplar as the biomass feedstock and pretreatment conditions reflecting the current state of technology, as demonstrated in bench-scale experiments (Table 1).

Equipment Number	Equipment Name	Equipment Sizing	Quantity	Unit Rate ($)	Purchasing Price ($)	Installed Cost ($)
GBX-109	Truck Scale	Rated Throughput: 17867.35 kg/h	1	32000	32000	54400
GBX-110	Truck Dumper	Rated Throughput: 17867.35 kg/h	1	140000	140000	238000
GBX-111	Truck Dumper Hopper	Rated Throughput: 17867.35 kg/h	1	145000	145000	246500
SL-101	Silo	Vessel Volume: 3923.81 m3	1	751000	751000	1276700
SR-103	Shredder	Rated Throughput: 5960 kg/h	3	127000	381000	571500
BC-107	Belt Conveyor	Belt Length: 19.81 m	1	4240000	4240000	7208000
BC-105	Belt Conveyor	Belt Length: 9.14 m	1	69000	69000	117300
VSCR-101	Vibrating Screen	Rated Throughput: 17867.35 kg/h	1	24000	24000	36000
SR-101	Shredder	Rated Throughput: 4720 kg/h	2	120000	240000	360000
BC-106	Belt Conveyor	Belt Length: 19.81 m	1	100000	100000	170000
BC-108	Belt Conveyor	Belt Length: 19.81 m	1	100000	100000	170000
GBX-101	Dome Reclaim System	Rated Throughput: 17867.35 kg/h	1	880000	880000	1320000
GBX-108	Belt Scale	Rated Throughput: 17867.35 kg/h	1	3000	3000	5100

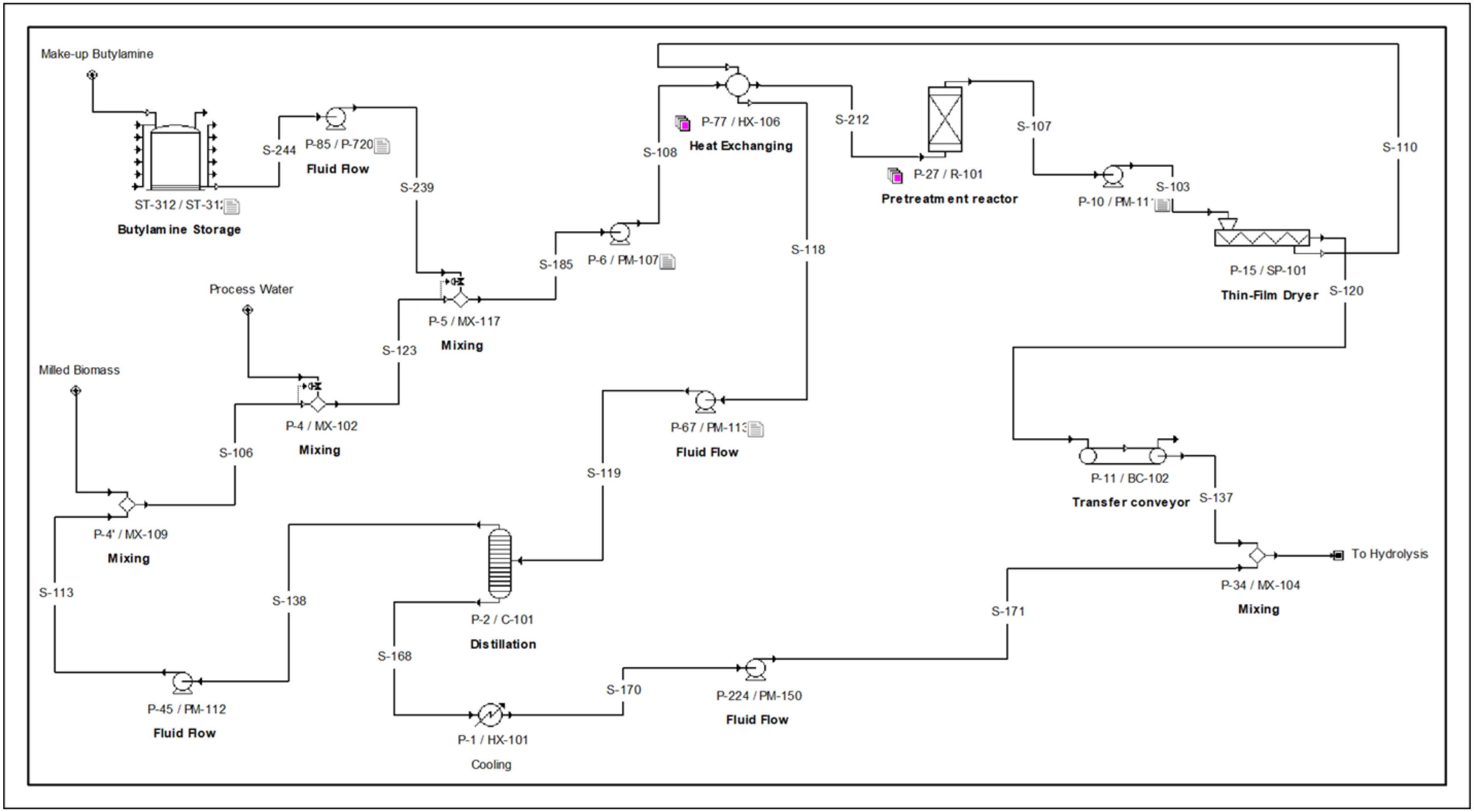

Figure S3. Process flow diagram of the biomass pretreatment and solvent recovery stages.

Table S6. Stream flow details associated with the biomass pretreatment and solvent recovery stages (see **Figure S3**). These are representative results based on poplar as the biomass feedstock and pretreatment conditions reflecting the current state of technology, as demonstrated in bench-scale experiments (Table 1).

Component	Units	Milled Biomass	S-106	Process Water	S-123	Make-up Butylamine	S-244	S-239	S-185	S-108	S-212
Total Mass Flow	kg/h	17867.4	23271.4	87696.1	110976.8	163.6	163.6	163.6	111138.4	111138.4	111138.4
Temperature	°C	25	40.9	25	26.8	25	25.1	25.1	26.8	26.8	100
Pressure	bar	1.01	1.01	1.01	1.01	1.01	1.01	1.57	1.01	1.57	1.57
Acetate	kg/h	706.7	706.7	0.0	706.7	0.0	0.0	0.0	706.7	706.7	706.7
Acetic-Acid	kg/h	0.0	0.0	0.0	0.0	0.0	0.0	0.0	0.0	0.0	0.0
Ash	kg/h	116.7	116.7	0.0	116.7	0.0	0.0	0.0	116.7	116.7	116.7
Butylamine	kg/h	0.0	5395.3	0.0	5395.3	163.6	163.6	163.6	5556.9	5556.9	5556.9
Cellulose	kg/h	7500.0	7500.0	0.0	7500.0	0.0	0.0	0.0	7500.0	7500.0	7500.0
Extractives	kg/h	709.8	709.8	0.0	709.8	0.0	0.0	0.0	709.8	709.8	709.8
Hemicellulose	kg/h	2800.0	2800.0	0.0	2800.0	0.0	0.0	0.0	2800.0	2800.0	2800.0
Lignin	kg/h	4833.3	4833.3	0.0	4833.3	0.0	0.0	0.0	4833.3	4833.3	4833.3
Protein	kg/h	0.2	0.2	0.0	0.2	0.0	0.0	0.0	0.2	0.2	0.2
Soluble lignin	kg/h	0.0	0.0	0.0	0.0	0.0	0.0	0.0	0.0	0.0	0.0
Water	kg/h	1200.7	1209.4	87696.1	88914.8	0.0	0.0	0.0	88914.8	88914.8	88914.8

Component	S-107	S-103	S-110	S-120	S-137	S-118	S-119	S-138	S-113	S-168	S-170	S-171	To Hydrolysis
Total Mass Flow	111138.4	111138.4	93270.1	17868.2	17868.2	93270.1	93270.1	5403.9	5403.9	87867.8	87867.8	87867.8	105734.4
Temperature	140	140	122	122	122	90	90	85.4	77.8	107.1	50	50	66
Pressure	4.10	4.10	2.10	2.10	2.10	1.59	2.14	1.30	2.30	1.30	1.30	1.32	2.10
Acetate	70.7	70.7	0.0	70.7	70.7	0.0	0.0	0.0	0.0	0.0	0.0	0.0	70.7
Acetic-Acid	636.0	636.0	577.3	58.7	58.7	577.3	577.3	0.0	0.0	577.3	577.3	577.3	636.0
Ash	116.7	116.7	0.0	116.7	116.7	0.0	0.0	0.0	0.0	0.0	0.0	0.0	116.7
Butylamine	5556.9	5556.9	5556.4	0.6	0.6	5556.4	5556.4	5395.2	5395.2	161.1	161.1	161.1	161.7
Cellulose	7500.0	7500.0	0.0	7500.0	7500.0	0.0	0.0	0.0	0.0	0.0	0.0	0.0	7500.0
Extractives	709.8	709.8	0.0	709.8	709.8	0.0	0.0	0.0	0.0	0.0	0.0	0.0	709.8
Hemicellulose	2800.0	2800.0	0.0	2800.0	2800.0	0.0	0.0	0.0	0.0	0.0	0.0	0.0	2800.0
Lignin	3866.7	3866.7	0.0	3866.7	3866.7	0.0	0.0	0.0	0.0	0.0	0.0	0.0	3866.7
Protein	0.2	0.2	0.0	0.2	0.2	0.0	0.0	0.0	0.0	0.0	0.0	0.0	0.2
Soluble lignin	966.7	966.7	0.0	966.7	966.7	0.0	0.0	0.0	0.0	0.0	0.0	0.0	966.7
Water	88914.8	88914.8	87136.5	1778.3	1778.3	87136.5	87136.5	8.7	8.7	80691.1	80691.1	80691.1	88906.1

Table S7. Equipment sizing and associated costs for the biomass pretreatment and solvent recovery stages (see **Figure S3**).

Equipment Number	Equipment Name	Equipment Sizing	Quantity	Unit Rate ($)	Purchasing Price ($)	Installed Cost ($)
ST-312	Flat Bottom Tank	Vessel Volume: 980 L	1	5500	5500	8250
P-720	Centrifugal Pump	Power: 0.01 kW	1	12000	12000	37200
PM-107	Centrifugal Pump	Power: 2.42 kW	1	35000	35000	108500
PM-111	Centrifugal Pump	Power: 0.01 kW	1	12000	12000	37200
PM-150	Centrifugal Pump	Pump Power: 3.54 kW	1	41000	41000	94300
C-101	Distillation Column	Column Volume: 5854.41 L	1	283000	283000	481100
PM-112	Centrifugal Pump	Pump Power: 0.3 kW	1	14000	14000	32200
PM-113	Centrifugal Pump	Power: 1.98 kW	1	32000	32000	99200
HX-106	Heat Exchanger	Heat Exchange Area: 95.43 m^2	8	154000	1232000	1848000
R-101	Pretreatment Reactor	Volume: 61.23 m^3	6	4863000	29178000	43767000
SP-101	Thin-Film Dryer	Throughput: 12340 kg/h	9	625000	5625000	9562500
HX-101	Heat Exchanger	Heat Exchange Area: 57.33 m^2	1	113000	113000	169500

S5. Sugar Yield and Butylamine Recovery at Varying Butylamine-To-Water Ratios

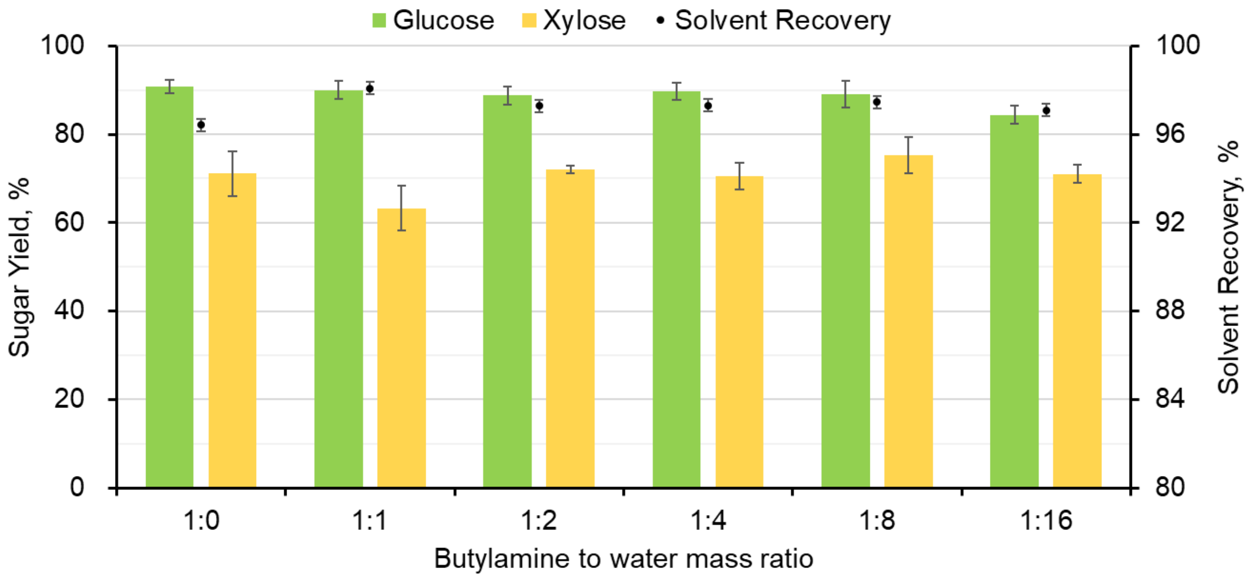

Figure S4. Sugar yield and butylamine recovery at varying butylamine-to-water ratios for poplar biomass feedstock (all data from our recent experimental work).[25]

S6. Sensitivity Analysis Results

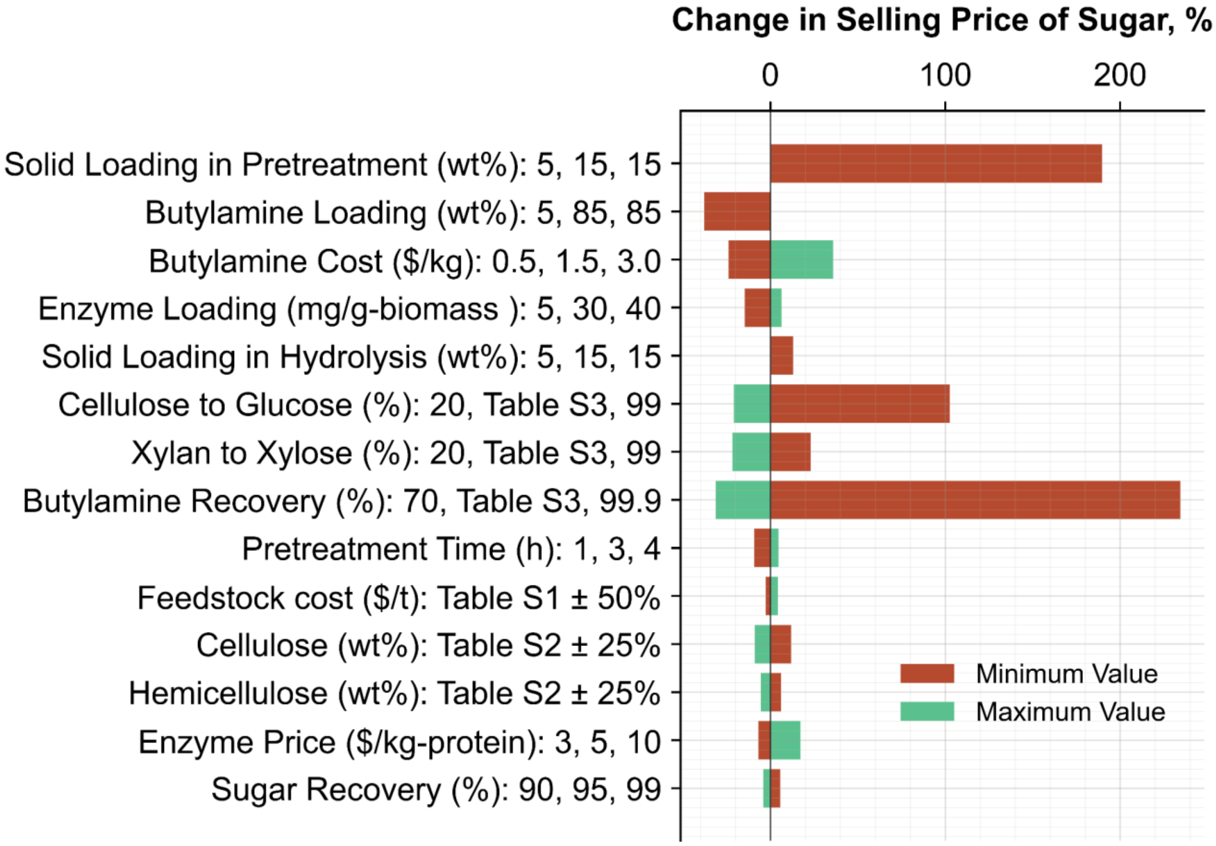

Figure S5. Key process parameters with the greatest influence on the minimum selling price of lignocellulosic sugar produced from **corn stover**.

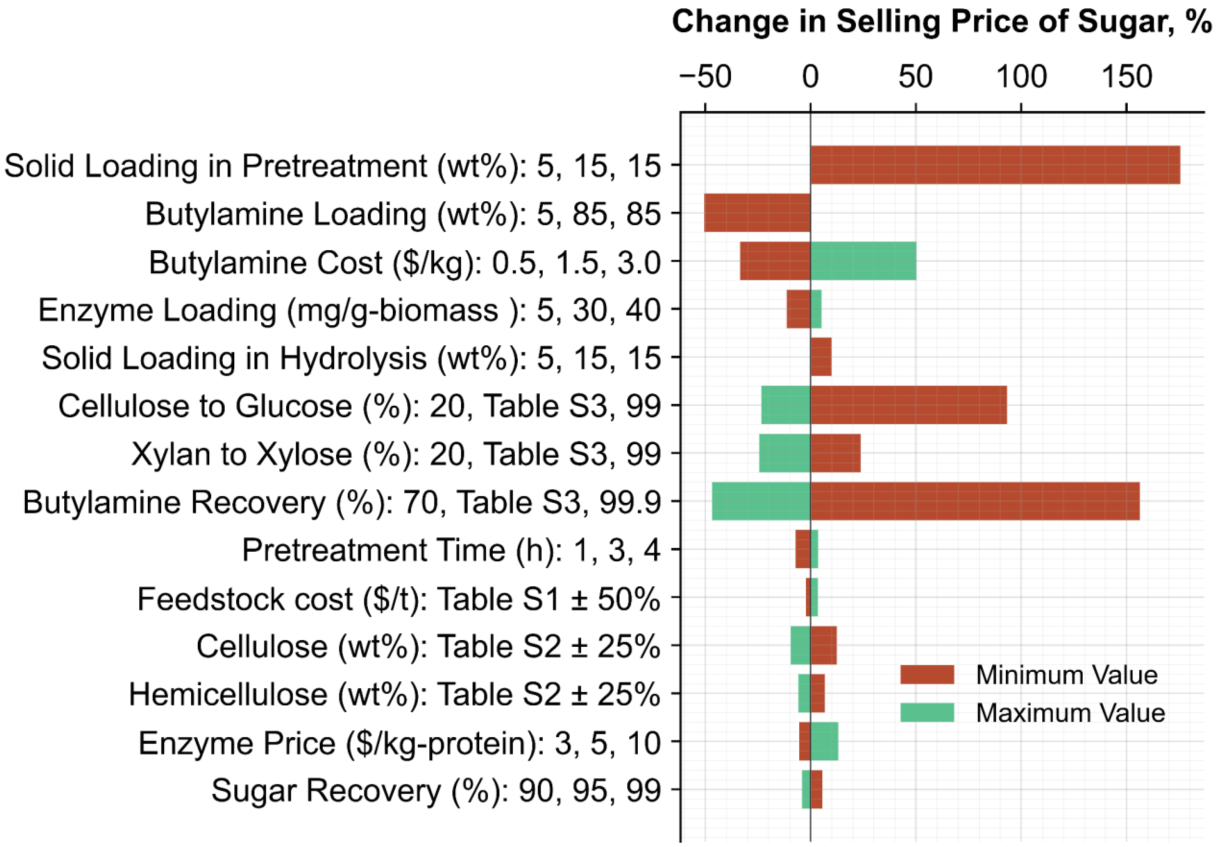

Figure S6. Key process parameters with the greatest influence on the minimum selling price of lignocellulosic sugar produced from **energy cane**.

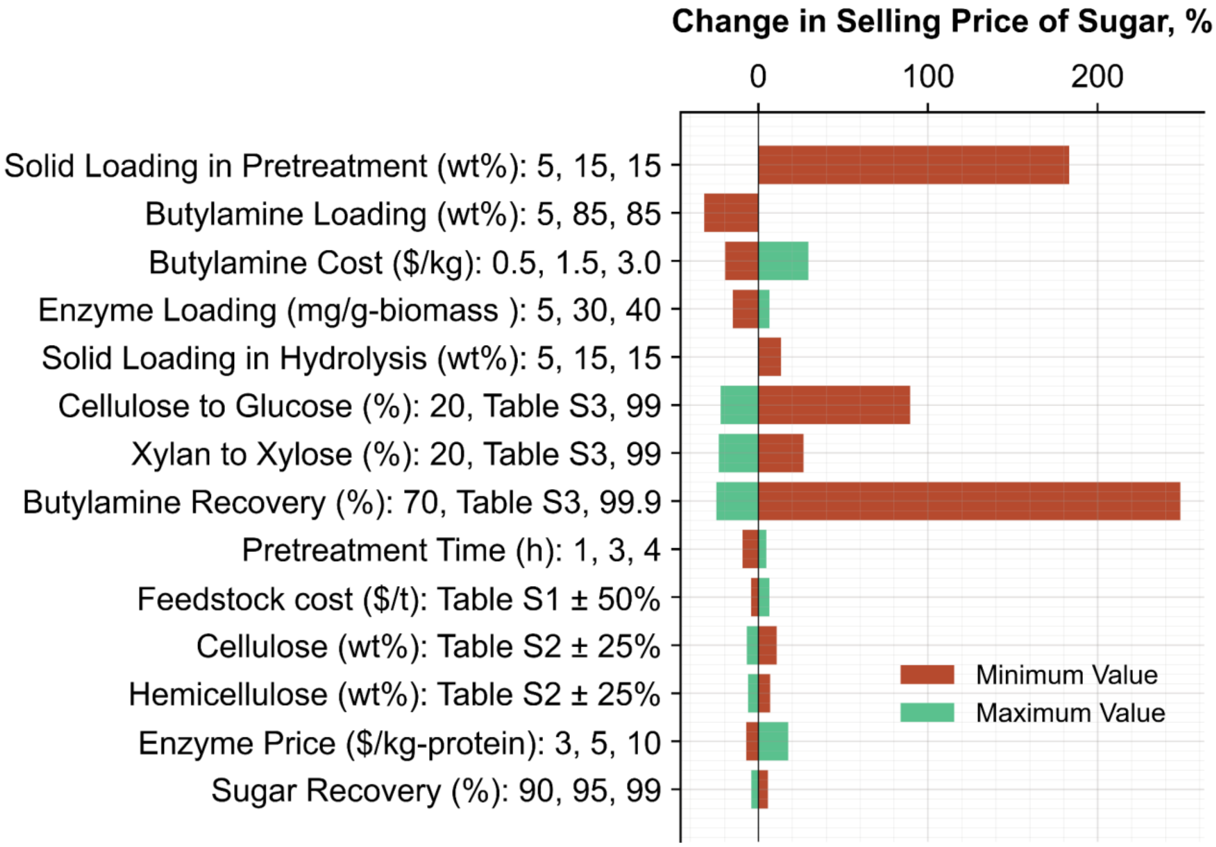

Figure S7. Key process parameters with the greatest influence on the minimum selling price of lignocellulosic sugar produced from **hay**.

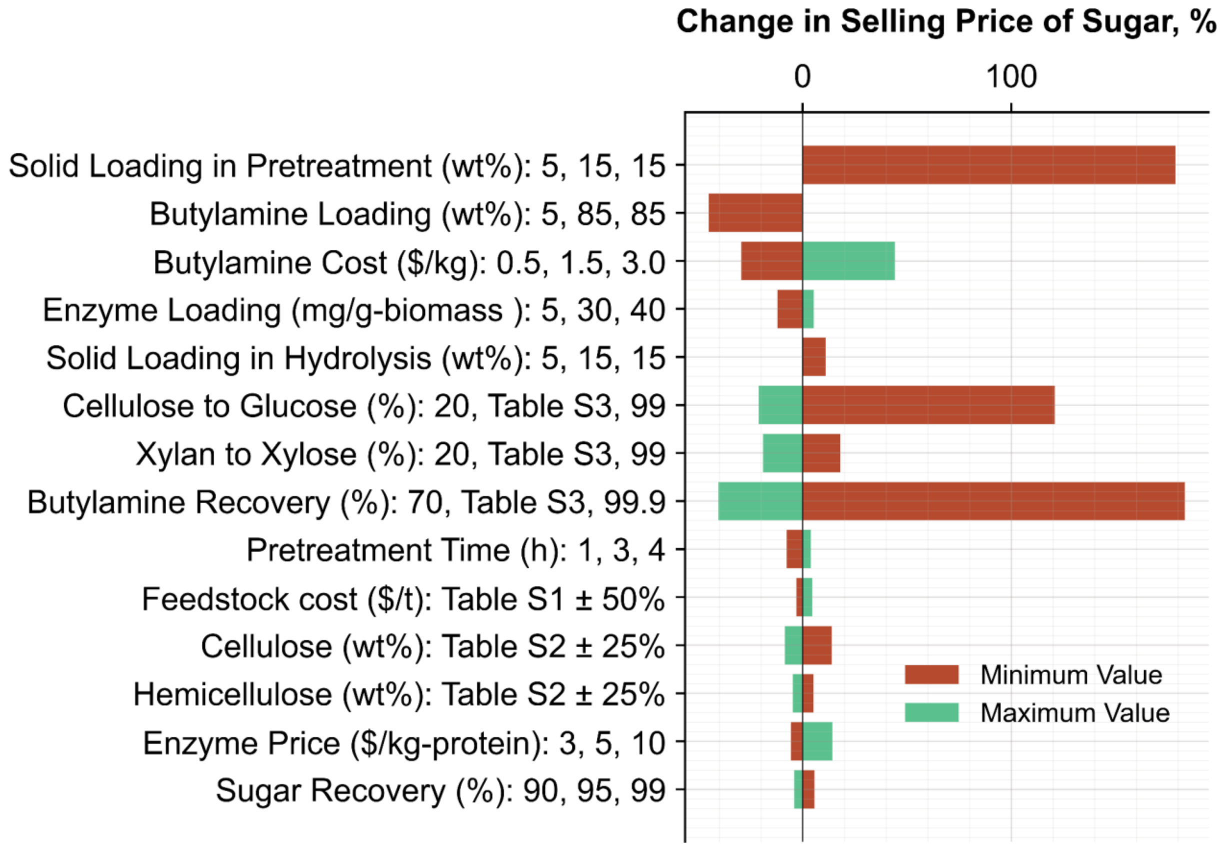

Figure S8. Key process parameters with the greatest influence on the minimum selling price of lignocellulosic sugar produced from **Miscanthus**.

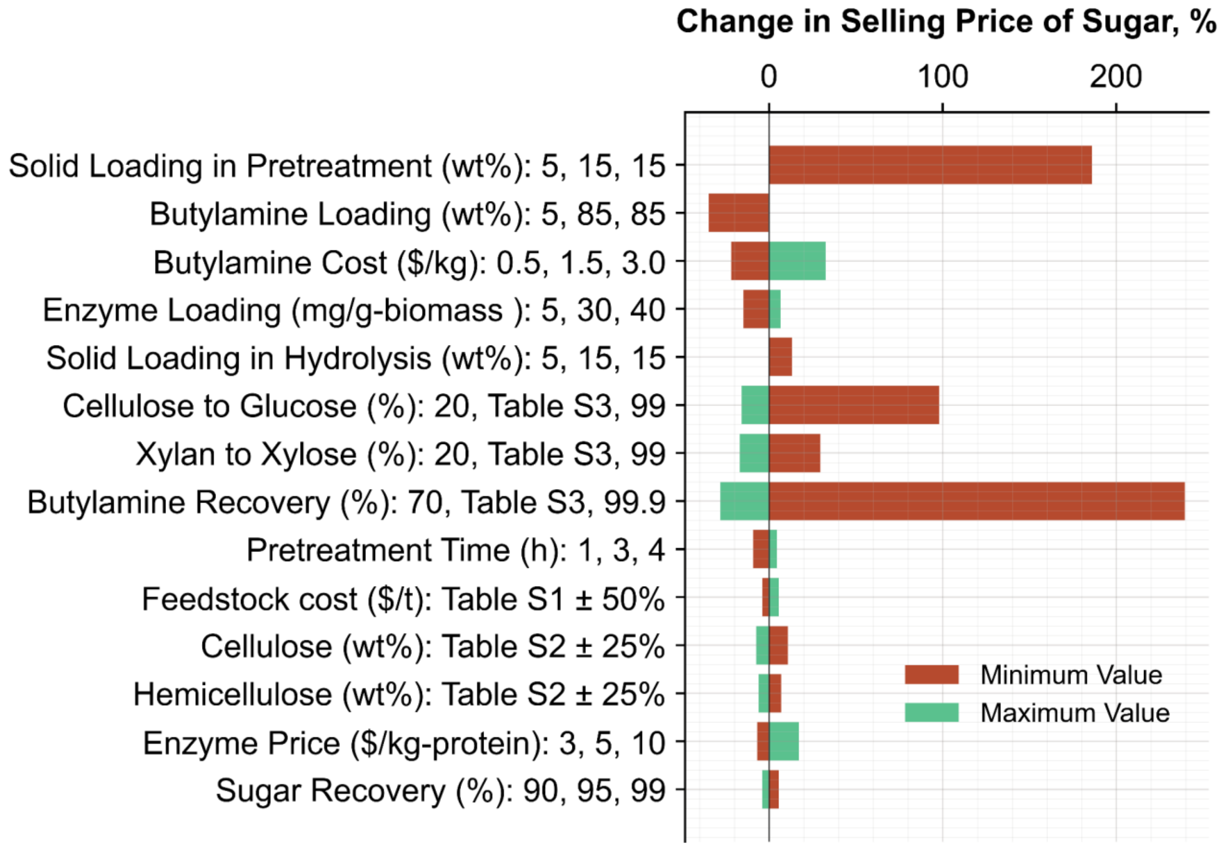

Figure S9. Key process parameters with the greatest influence on the minimum selling price of lignocellulosic sugar produced from **biomass sorghum**.

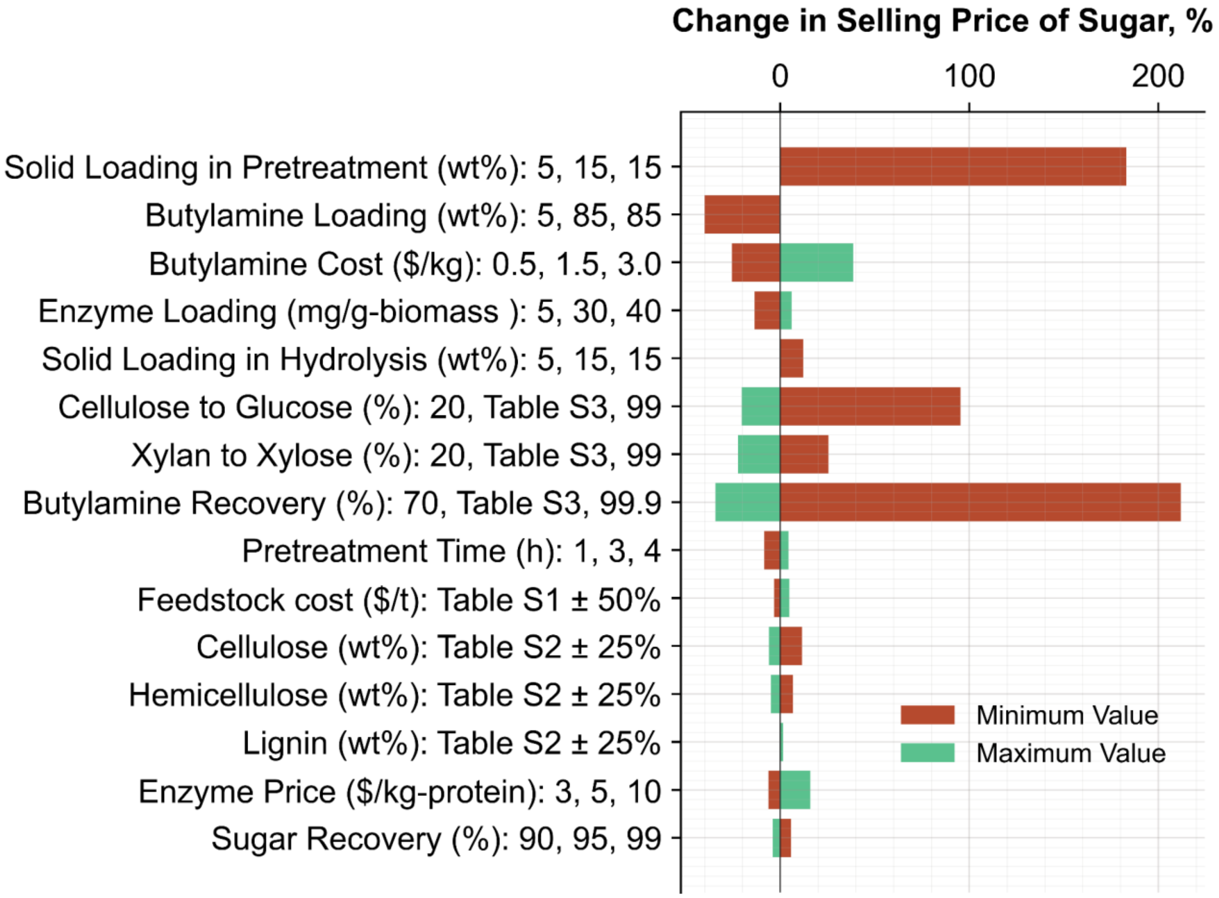

Figure S10. Key process parameters with the greatest influence on the minimum selling price of lignocellulosic sugar produced from **switchgrass**.

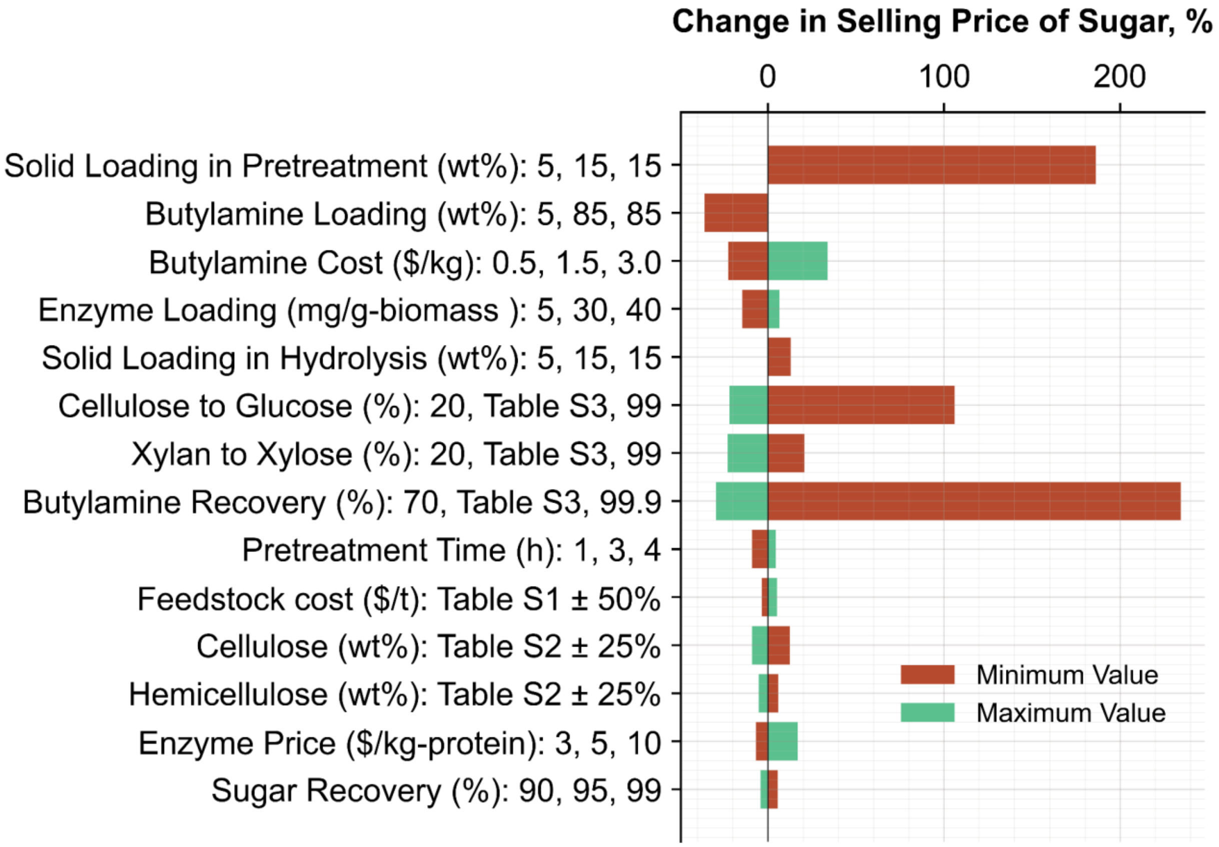

Figure S11. Key process parameters with the greatest influence on the minimum selling price of lignocellulosic sugar produced from **wheat straw**.

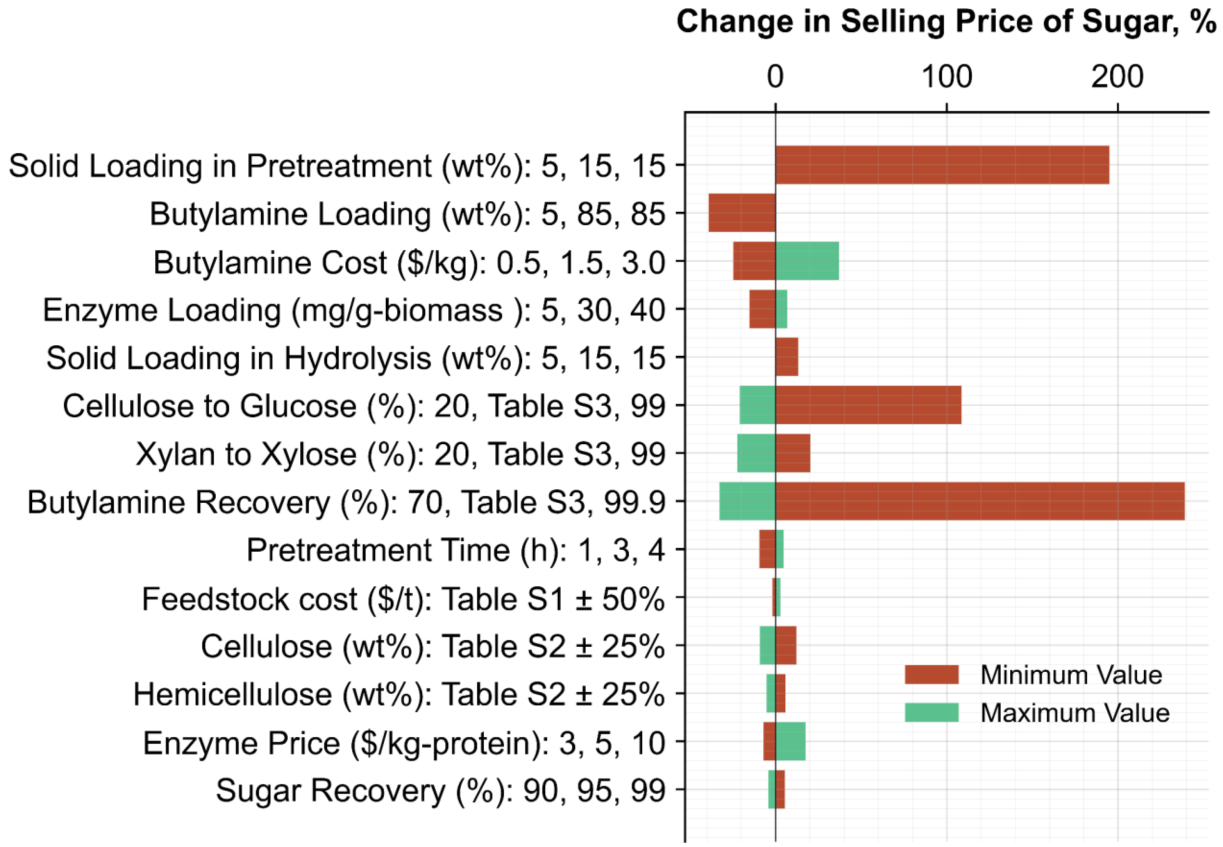

Figure S12. Key process parameters with the greatest influence on the minimum selling price of lignocellulosic sugar produced from **almond stems**.

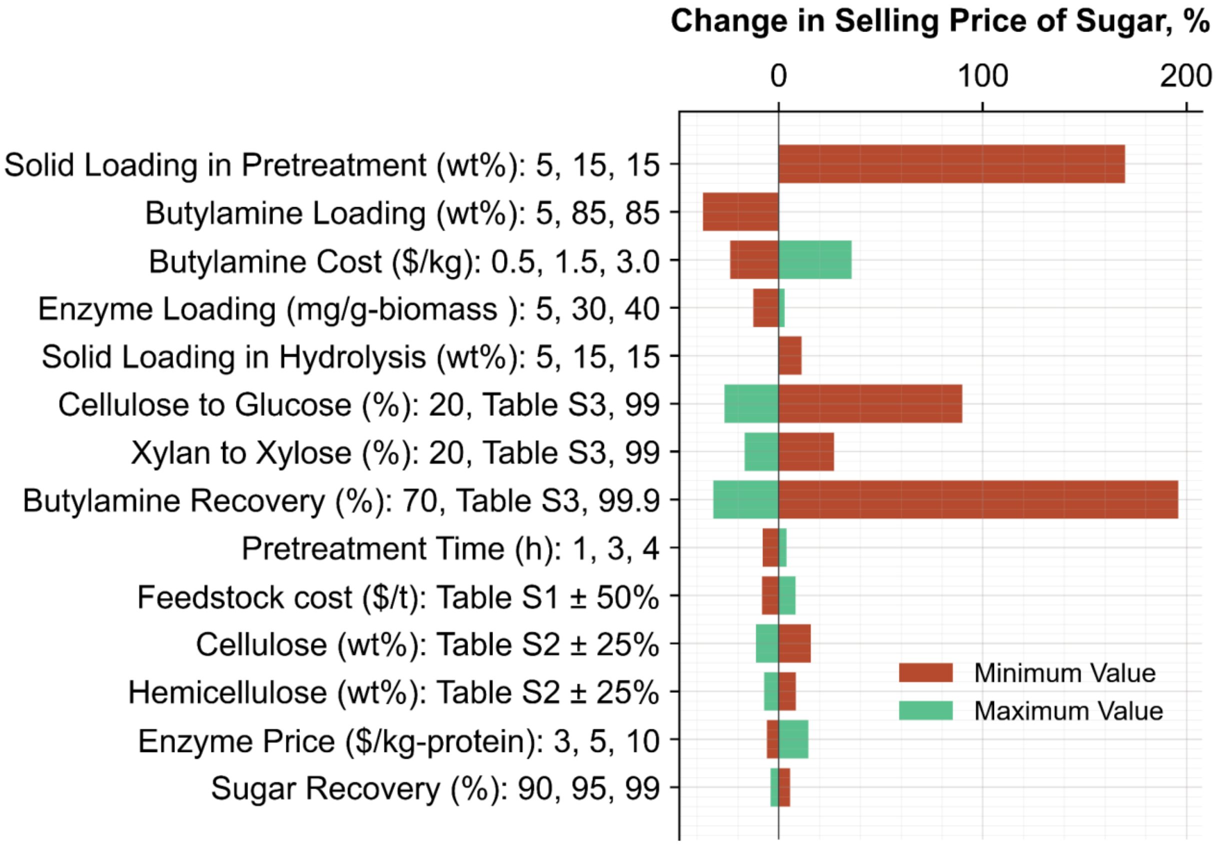

Figure S13. Key process parameters with the greatest influence on the minimum selling price of lignocellulosic sugar produced from **bamboo sticks**.

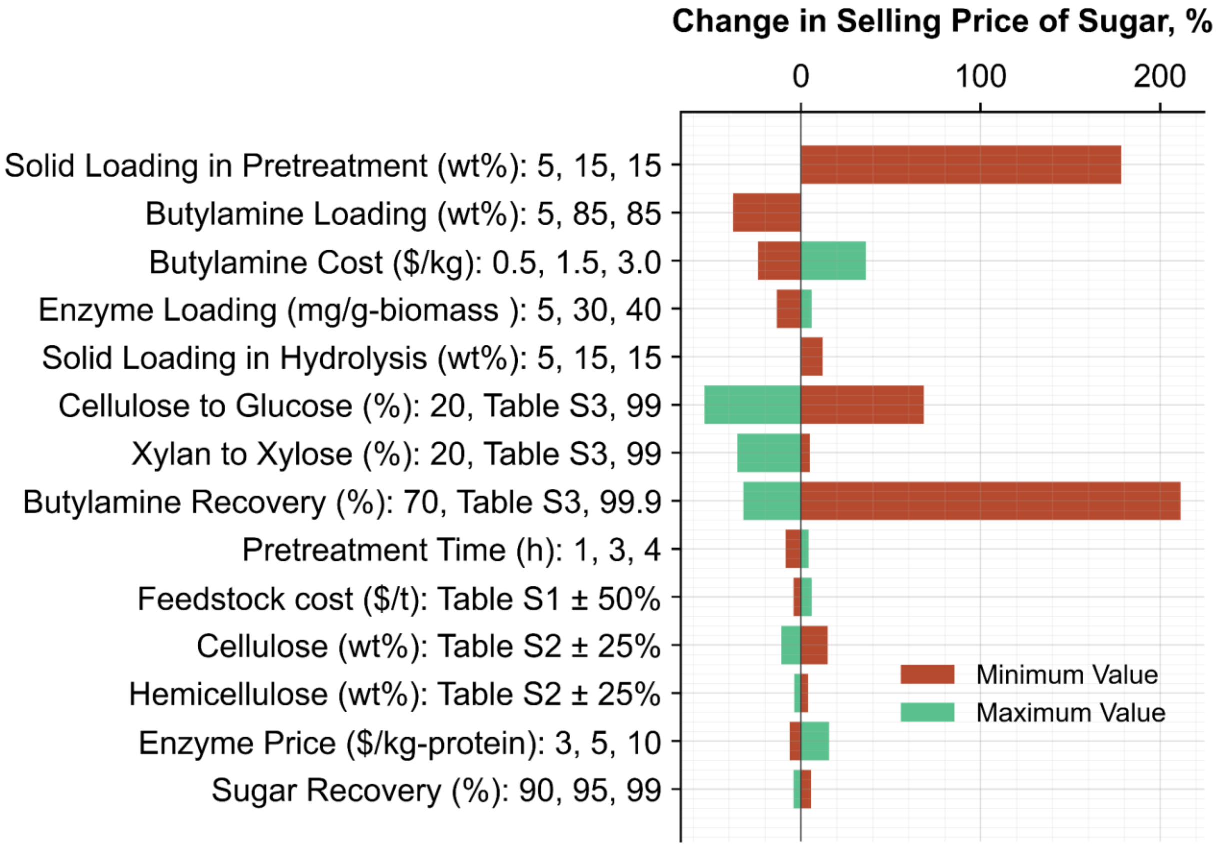

Figure S14. Key process parameters with the greatest influence on the minimum selling price of lignocellulosic sugar produced from **eucalyptus**.

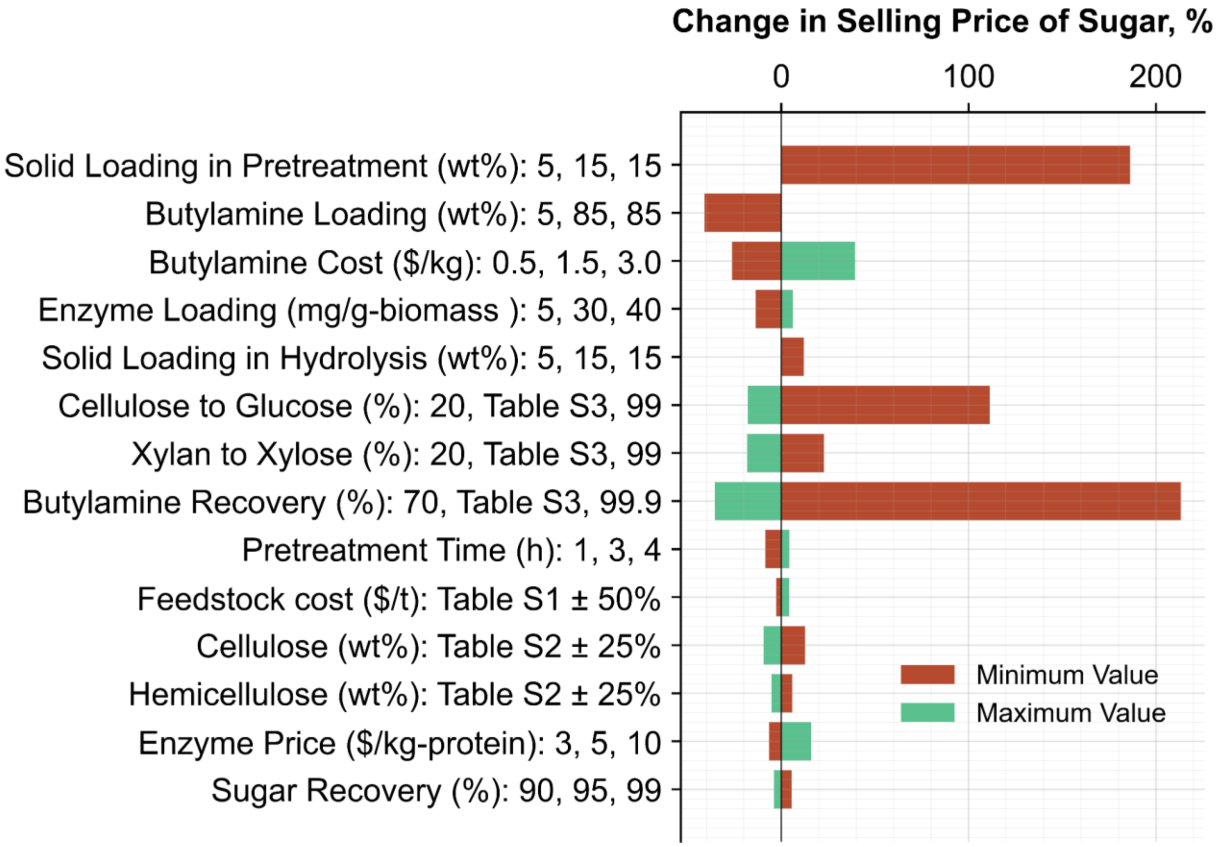

Figure S15. Key process parameters with the greatest influence on the minimum selling price of lignocellulosic sugar produced from **hardwood sawdust 1**.

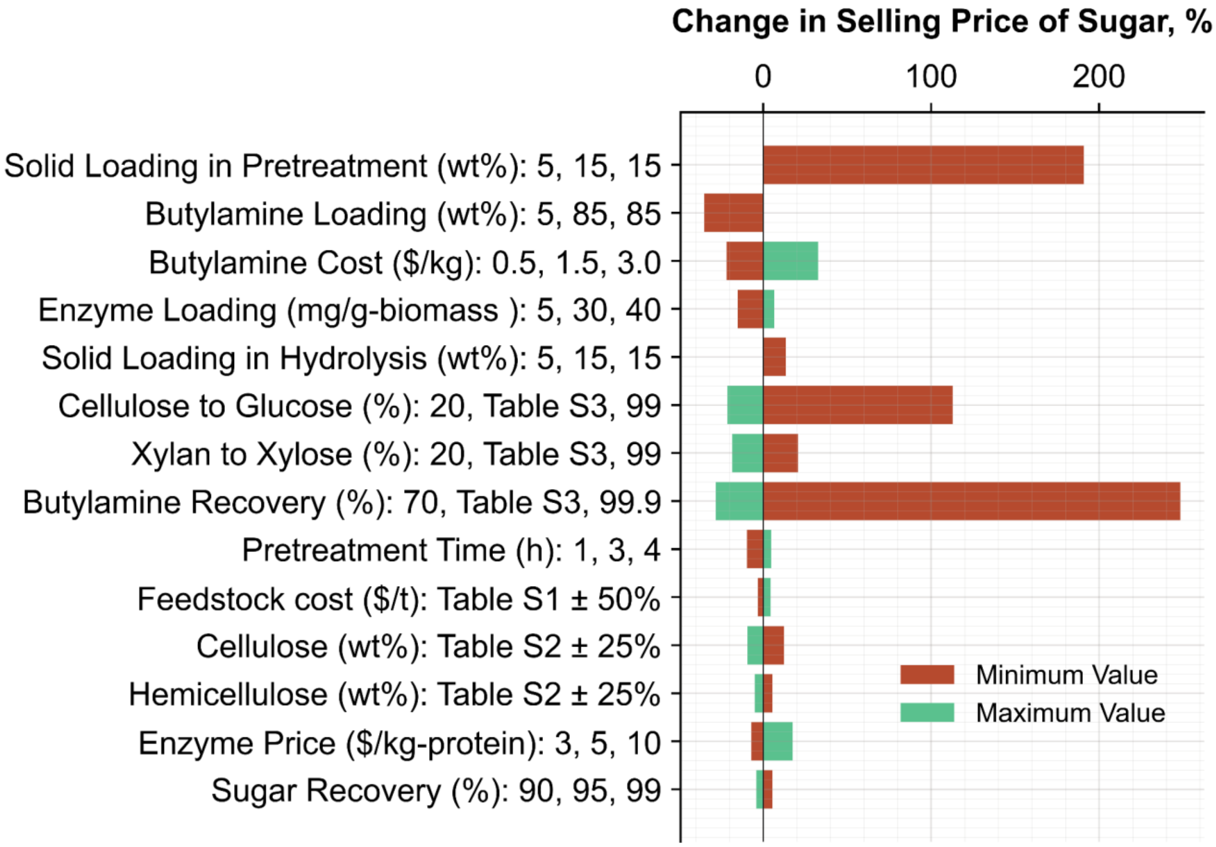

Figure S16. Key process parameters with the greatest influence on the minimum selling price of lignocellulosic sugar produced from **hardwood sawdust 2**.

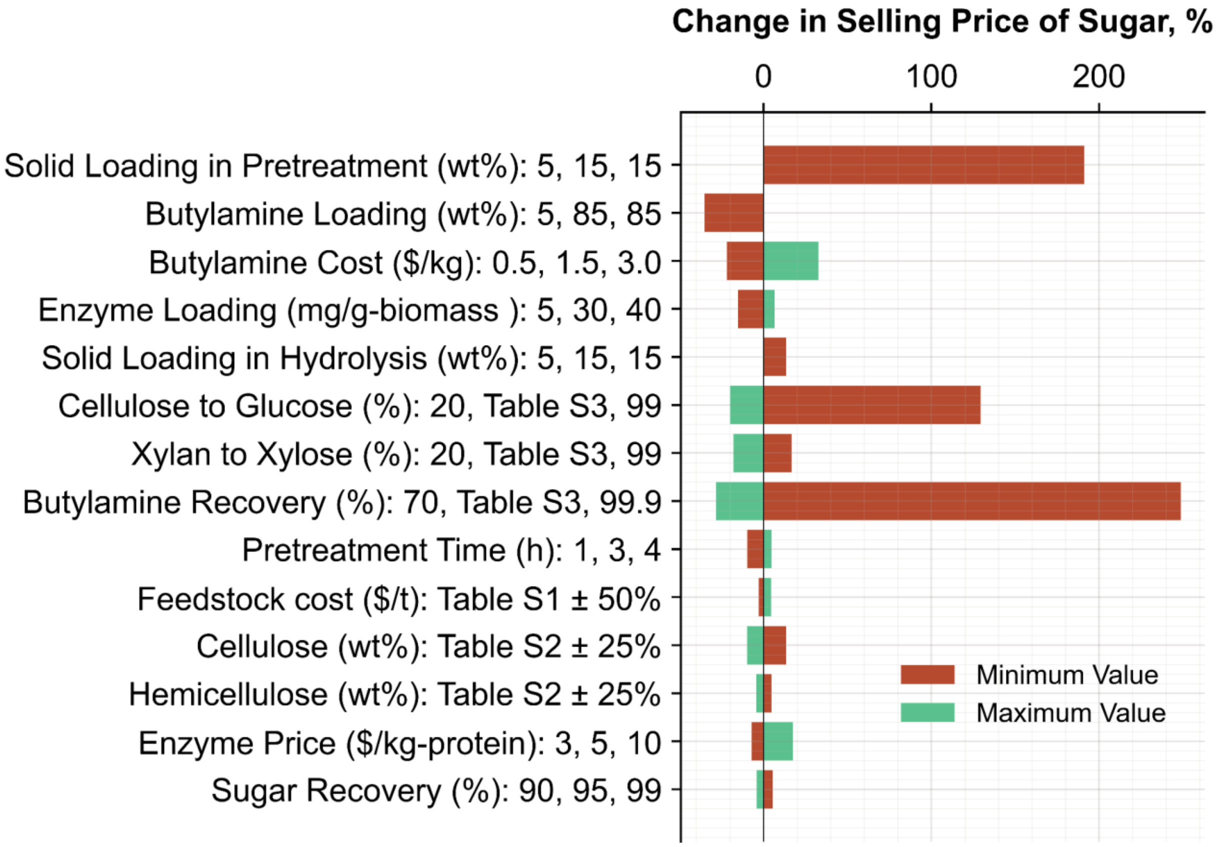

Figure S17. Key process parameters with the greatest influence on the minimum selling price of lignocellulosic sugar produced from **hardwood sawdust 3**.

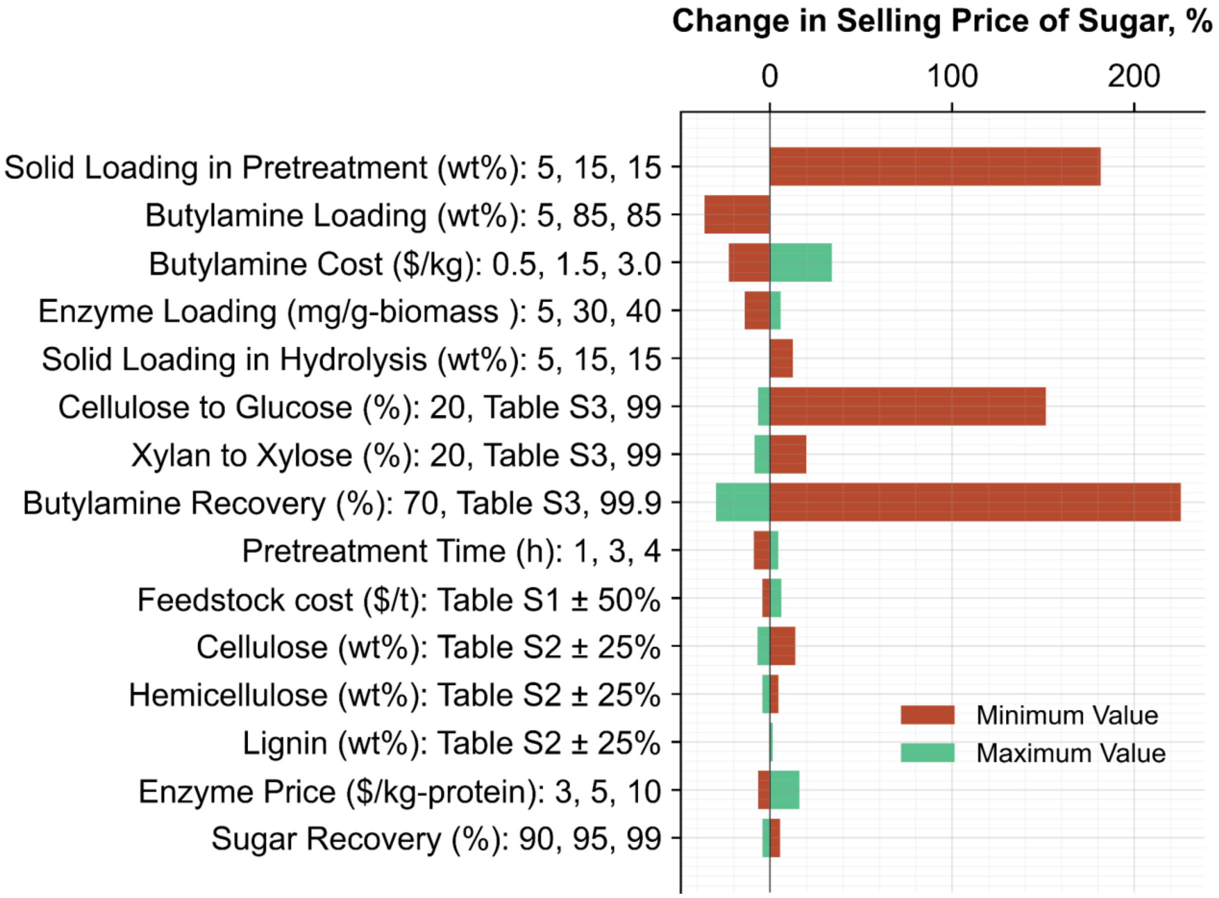

Figure S18. Key process parameters with the greatest influence on the minimum selling price of lignocellulosic sugar produced from **poplar**.

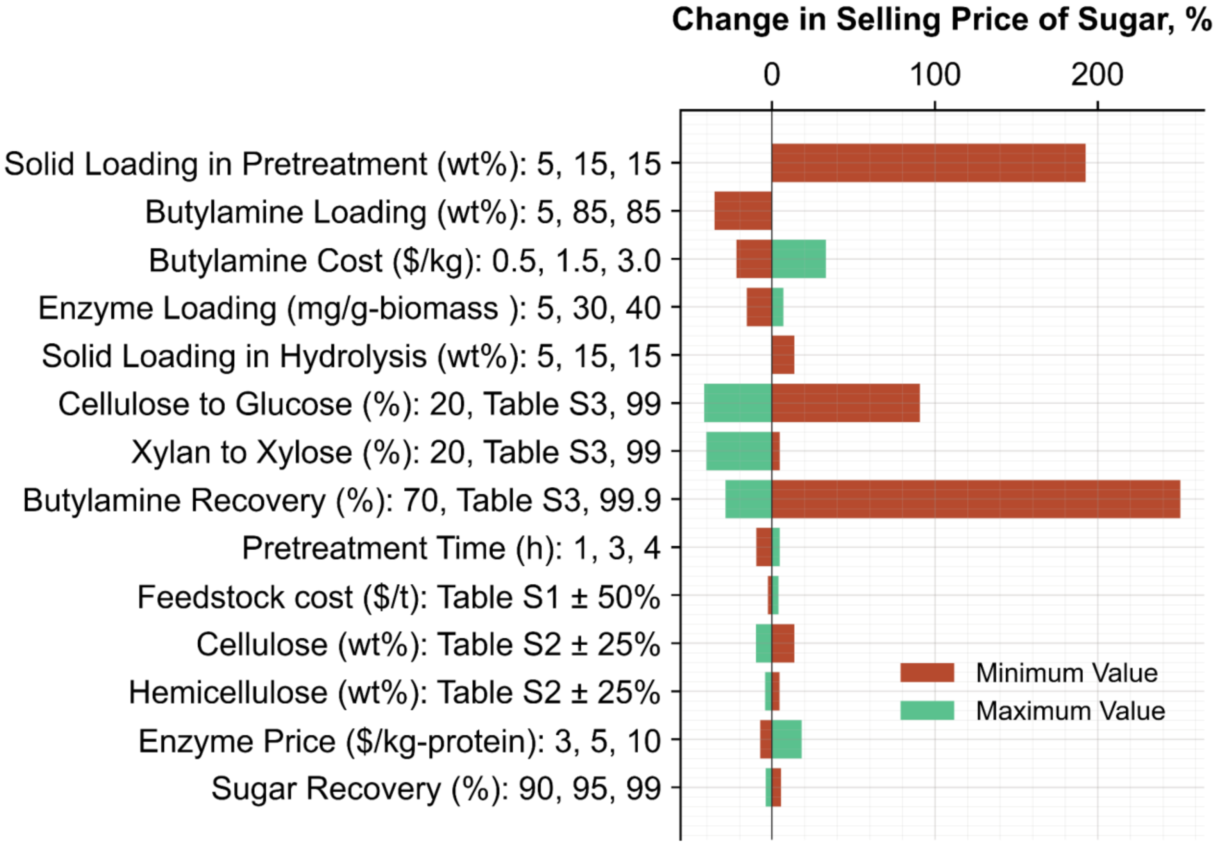

Figure S19. Key process parameters with the greatest influence on the minimum selling price of lignocellulosic sugar produced from **pine**.

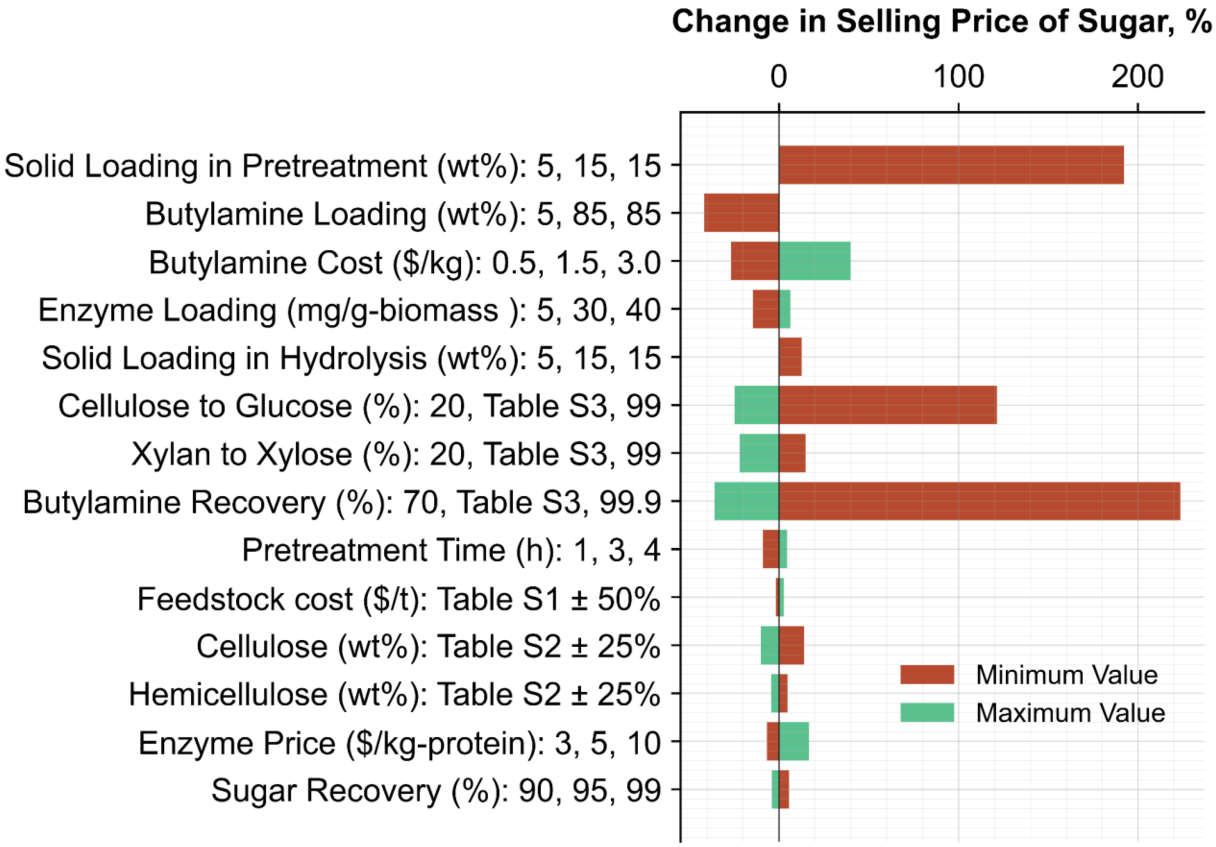

Figure S20. Key process parameters with the greatest influence on the minimum selling price of lignocellulosic sugar produced from **walnut stems**.

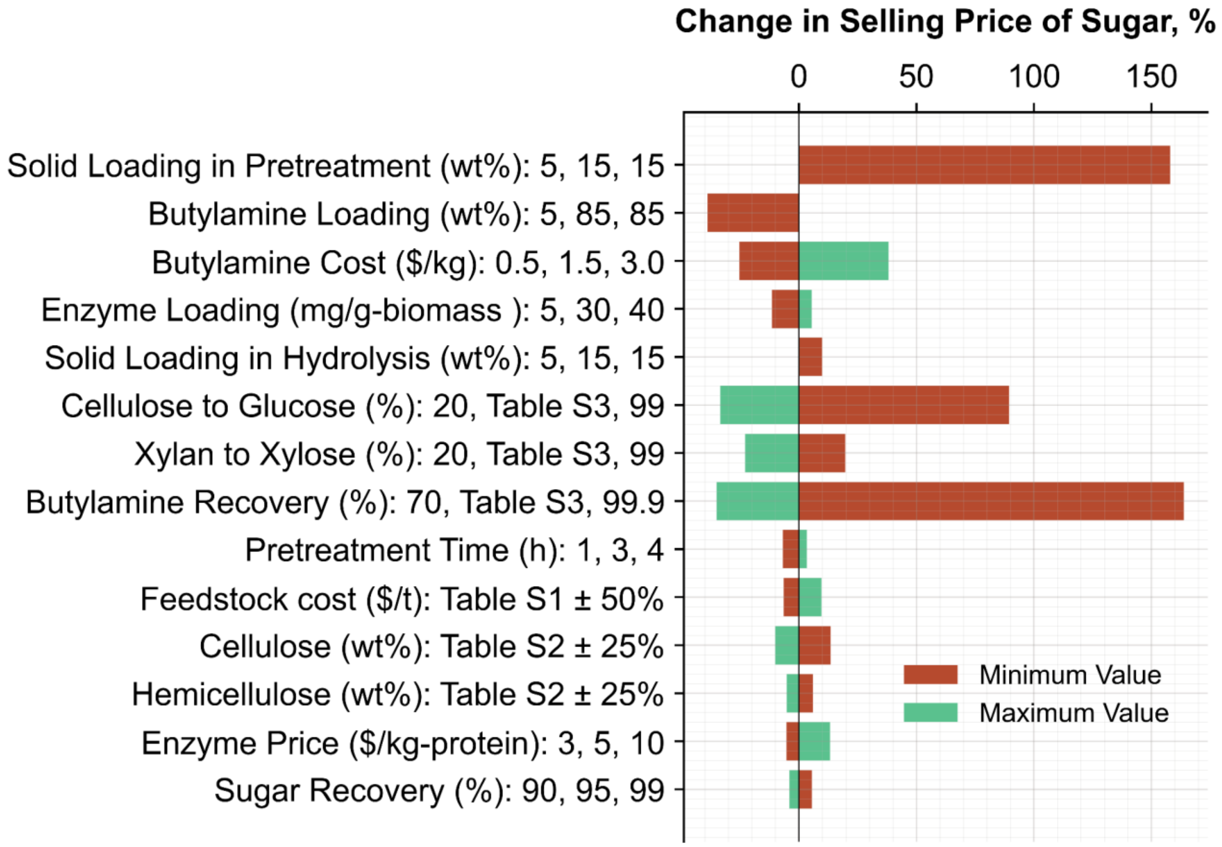

Figure S21. Key process parameters with the greatest influence on the minimum selling price of lignocellulosic sugar produced from **coconut chips**.

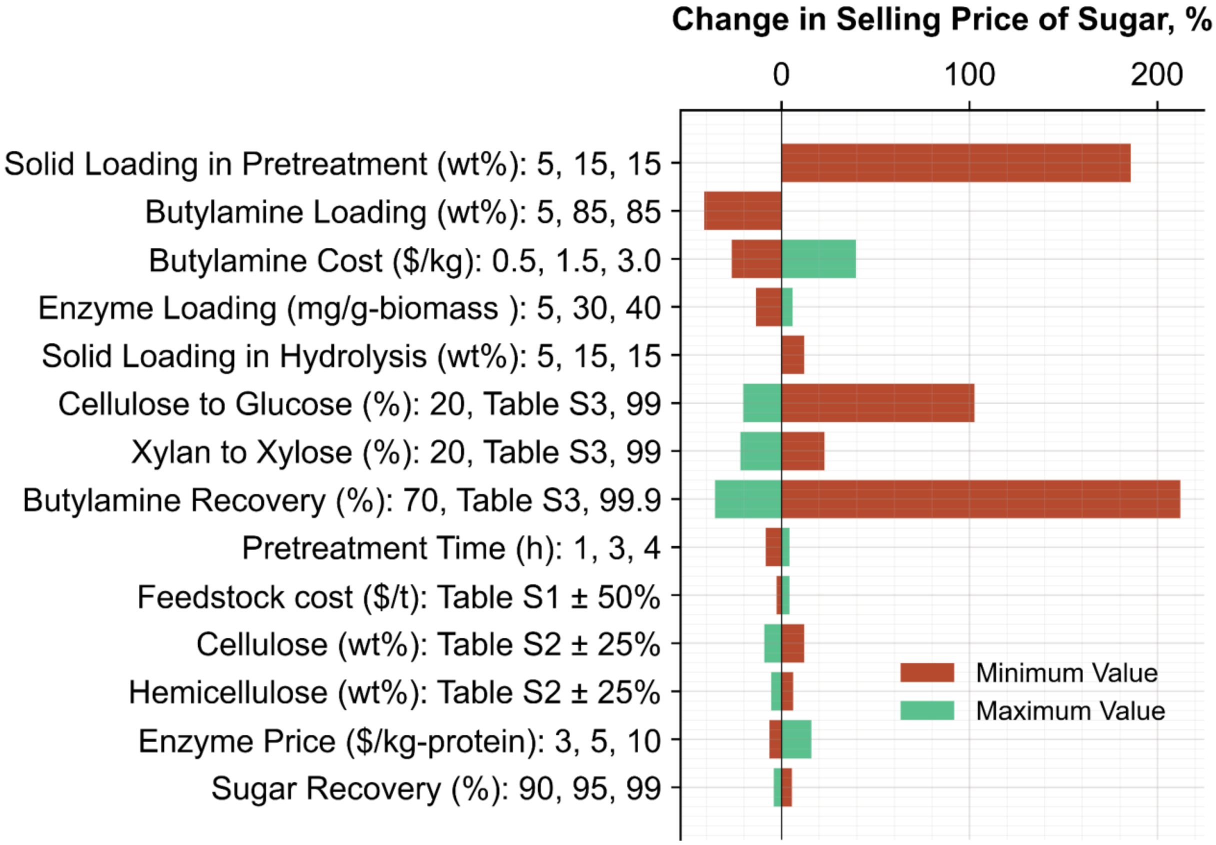

Figure S22. Key process parameters with the greatest influence on the minimum selling price of lignocellulosic sugar produced from **sugarcane bagasse**.

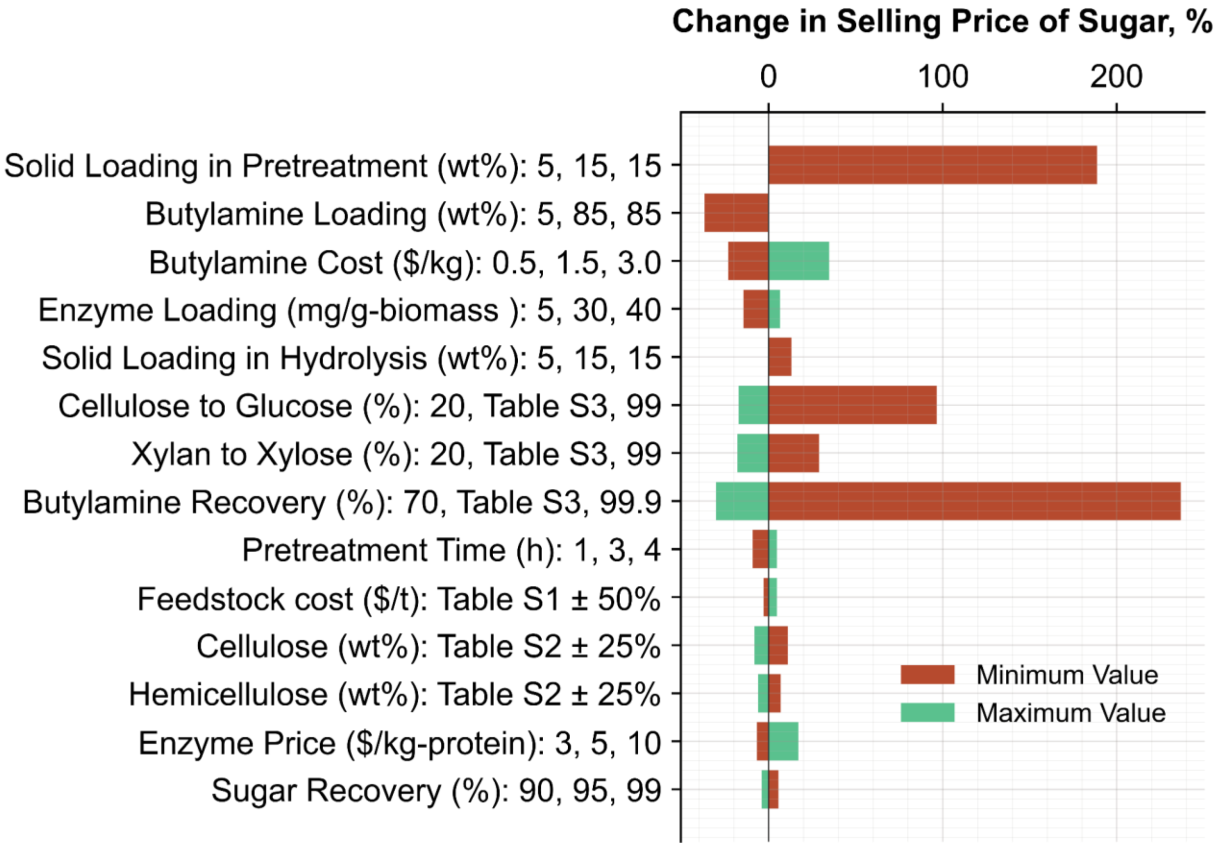

Figure S23. Key process parameters with the greatest influence on the minimum selling price of lignocellulosic sugar produced from **oil palm fiber**.

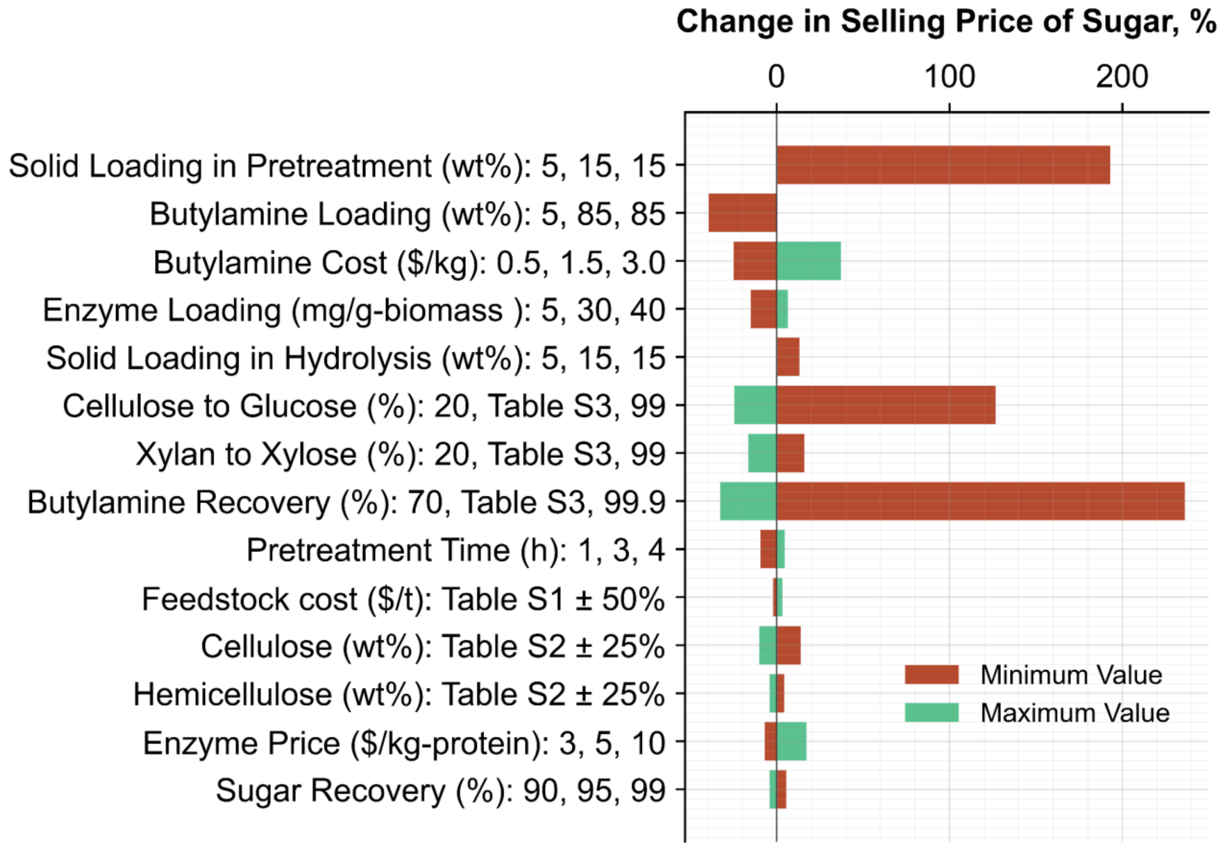

Figure S24. Key process parameters with the greatest influence on the minimum selling price of lignocellulosic sugar produced from **rice hulls**.

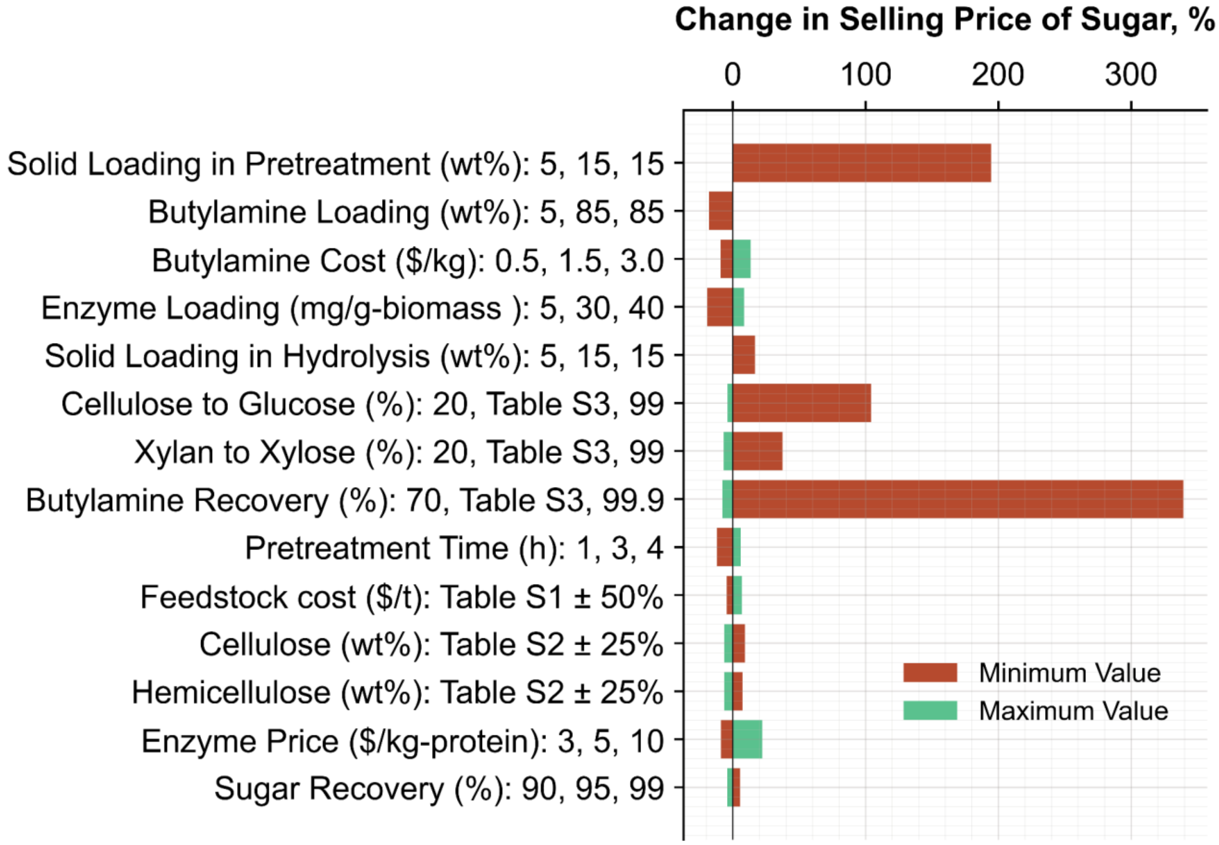

Figure S25. Key process parameters with the greatest influence on the minimum selling price of lignocellulosic sugar produced from **herbaceous blend**.

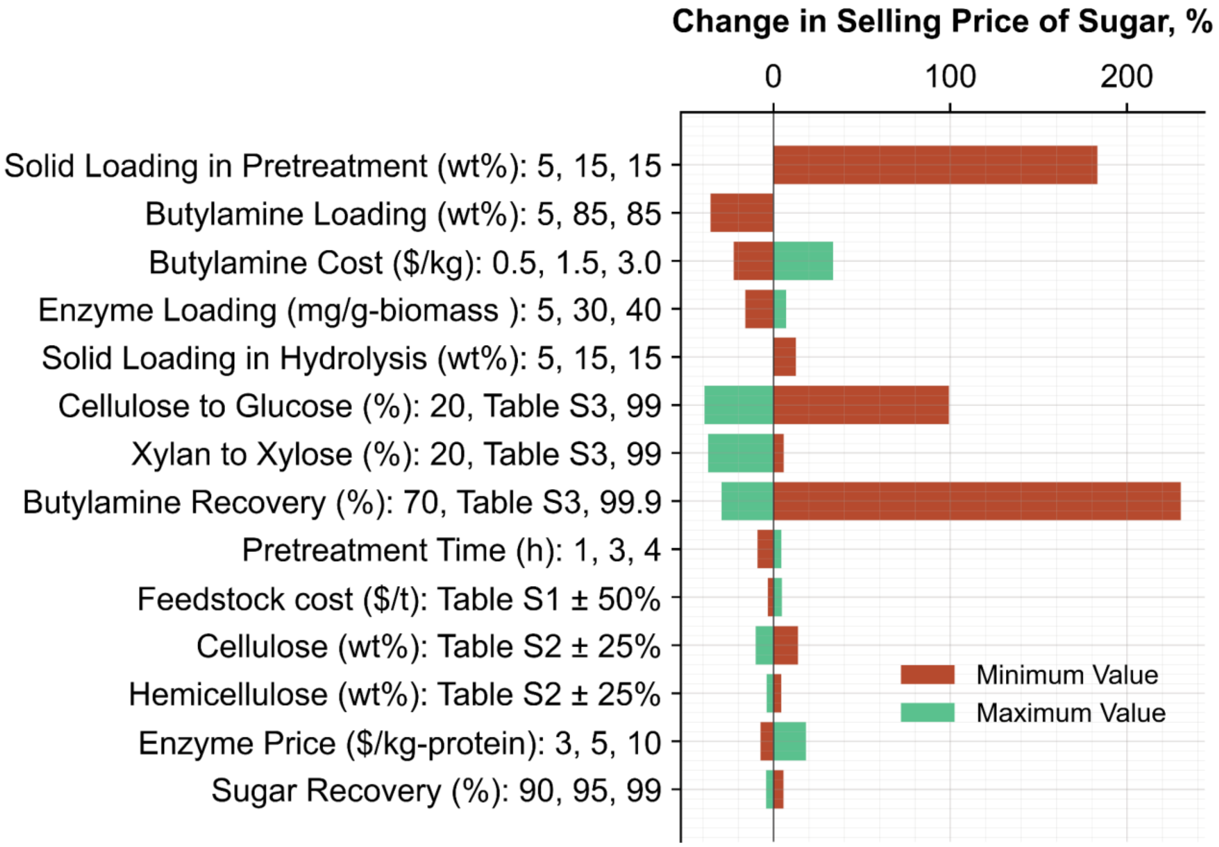

Figure S26. Key process parameters with the greatest influence on the minimum selling price of lignocellulosic sugar produced from **agri-woody blend**.

S7. Progressive Reduction in the Minimum Selling Price of Sugar

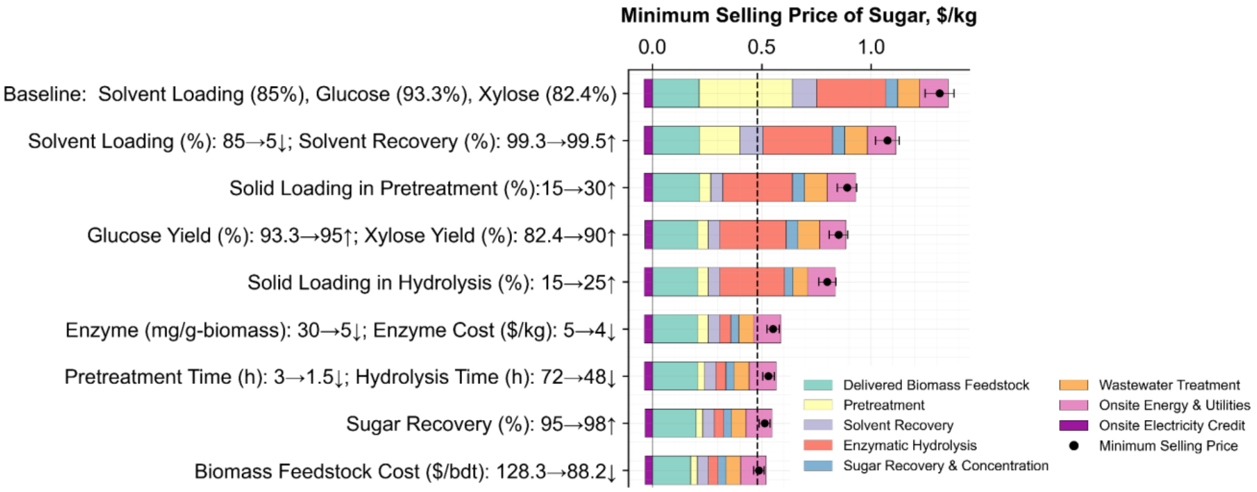

Figure S27. Stepwise decrease in the minimum selling price of sugar using a **herbaceous blend**—comprising equal amounts of corn stover, biomass sorghum, hay, and wheat straw—as the representative feedstock.

References

(1) Baral, N. R.; Dahlberg, J.; Putnam, D.; Mortimer, J. C.; Scown, C. D. Supply cost and life-cycle greenhouse gas footprint of dry and ensiled biomass sorghum for biofuel production. *ACS Sustain. Chem. Eng.* **2020**, *8*, 15855–15864.

(2) Baral, N. R.; Davis, R.; Bradley, T. H. Supply and value chain analysis of mixed biomass feedstock supply system for lignocellulosic sugar production. *Biofuels, Bioprod. Bioref.* **2019**, *13*, 635–659.

(3) U.S. Department of Energy. 2023 Billion-Ton Report, **2024**, https://www.energy.gov/sites/default/files/2023-06/beto-01-energy-crops-saf-panel-june-2023-langholtz.pdf (accessed Jan 2, 2024).

(4) United States Department of Agriculture (USDA), **2025**, https://www.ams.usda.gov/mnreports/ams_2904.pdf (accessed May 1, 2025).

(5) United States Department of Agriculture (USDA), **2025**, https://www.ams.usda.gov/mnreports/ams_2243.pdf (accessed May 1, 2025).

(6) Hull Forest Products. Bulk Sawdust Pickup & Delivery Prices, **2025**, https://www.hullforest.com/sawmill/sawdust-wholesale-retail-mill-direct-connecticut-delivery-available/ (accessed May 2, 2025).

(7) United States Department of Agriculture (USDA), **2025**, https://www.ams.usda.gov/mnreports/ams_1655.pdf (accessed May 2, 2025).

(8) University of Missouri Extension. All By-Products, Sorted by Company, **2025**, http://agebb.missouri.edu/dairy/byprod/allcompanies.asp (accessed May 2, 2025).

(9) Eisenbies, M.; Volk, T.; Amidon, T.; Bergey, S.; Bold-Erdene, Z.; Clark, R.; DeSouza, D.; Ebadian, M.; Emerson, R.; Gantz, C. Improved Advanced Biomass Logistics Utilizing Woody and other Feedstocks in the Northeast and Pacific Northwest; State Univ. of New York (SUNY), Syracuse, NY, **2020**, https://www.osti.gov/servlets/purl/1768177 (accessed May 15, 2025).

(10) Jacobson, J. J., Roni, M. S., Lamers, P., & Cafferty, K. G. Biomass feedstock and conversion supply system design and analysis (No. INL/EXT-14-32377). Idaho National Laboratory (INL), Idaho Falls, ID, **2014**, https://inldigitallibrary.inl.gov/sites/sti/sti/6359707.pdf (accessed May 15, 2025).

(11) Paper Index. Wood Chip Listing, **2025**, https://www.paperindex.com/product-listings/wood-chips/18614/21 (accessed May 2, 2025).

(12) Burli, P. H.; Lin, Y.; Hartley, D. S.; Thompson, D. N. Woody Feedstocks 2022 State of Technology Report; Idaho National Laboratory (INL), Idaho Falls, ID, **2024**, https://inldigitallibrary.inl.gov/sites/sti/sti/Sort_63795.pdf (accessed May 15, 2025)

(13) Biodegradable and Compostable Pots. Coconut Fiber Price, Palm Fiber Price & Kenaf Fiber Price, **2025**, https://biodegradable-pots.com/coconut-mat-price/ (accessed May 2, 2025).

(14) Vietnam Washed Coco Husk Chips, **2025**, https://www.alibaba.com/product-detail/Vietnam-Washed-Coco-Husk-Chips-for_50035014104.html?spm=a2700.7724857.0.0.1de528e4ZA9ASm (accessed May 2, 2025).

(15) MSU Extension. What's the nutrient value of wheat straw? **2024**, https://www.canr.msu.edu/news/whats_the_nutrient_value_of_wheat_straw (accessed May 2, 2025).

(16) University of Minnesota. The Value of Wheat Straw, **2024**, https://blog-crop-news.extension.umn.edu/2020/07/the-value-of-wheat-straw.html?m=1 (accessed May 2, 2025).

(17) NDSU Agriculture. The Fertilizer Value of Wheat Straw, **2017**, https://www.ndsu.edu/agriculture/ag-hub/ag-topics/crop-production/soil-health/organic-matter-soil/fertilizer-value-wheat-straw (accessed May 2, 2025).

(18) Leal Silva, J. F.; Grekin, R.; Mariano, A. P.; Maciel Filho, R. Making levulinic acid and ethyl levulinate economically viable: A worldwide technoeconomic and environmental assessment of possible routes. *Energy Technology* **2018**, *6*, 613–639.

(19) Gadkari, S.; Narisetty, V.; Maity, S. K.; Manyar, H.; Mohanty, K.; Jeyakumar, R. B.; Pant, K. K.; Kumar, V. Techno-Economic Analysis of 2,3-Butanediol Production from Sugarcane Bagasse. *ACS Sustain. Chem. Eng.* **2023**, *11*, 8337–8349.

(20) Chang, S.J. To burn or not to burn: Bagasse, **2015**, https://www.swst.org/wp/meetings/AM15/pdfs/presentations/chang.pdf (accessed May 18, 2025).

(21) EIA. United States Natural Gas Industrial Price, **2025**, https://www.eia.gov/dnav/ng/hist/n3035us3m.htm (accessed May 18, 2025).

(22) University of Florida. Evaluation of Energycane for Bioenergy and Sustainable Agricultural Systems, **2023**, https://www.energy.gov/sites/default/files/2023-05/beto-11-project-peer-review-dma-apr-2023-sandhu.pdf (accessed May 2, 2025).

(23) Chen, X.; Krishnamoorthy, A.; Palasz, J.; Pidatala, V. R.; Lewis, T.; Tian, Y.; Barcelo, C.; Zhou, X.; Kang, X.; Dou, C.; Choudhary, H.; Sun, N.; Sundstrom, E.; Eudes, A.; Simmons, B. A. Distillable Amine-Based Solvents for Effective Pretreatment of Multiple Biomass Feedstocks. 2025, Available at SSRN 5138993.

(24) Li, Y.; Tao, L.; Nagle, N.; Tucker, M.; Chen, X.; Kuhn, E. M. Effect of feedstock variability, feedstock blends, and pretreatment conditions on sugar yield and production costs. *Front. Energy Res.* **2022**, *9*.

(25) Palasz, J. M.; Krishnamoorthy, A.; Giovine, R.; Chen, X.; Pidatala, V.; Turumtay, E. A.; Lewis, T.; Baidoo, E. E. K.; Dou, C.; Choudhary, H.; Simmons, B. A. The

Importance of Ester Cleavage in the Butylamine Pretreatment of Hybrid Poplar. Green Chem. 2025, DOI: 10.1039/D5GC01795E.

(26) Harrison, T. J. Development of the Mathematics of Learning Curve Models for Evaluating Small Modular Reactor Economics. Oak Ridge National Laboratory, Oak Ridge, TN (United States), 2014, https://www.osti.gov/servlets/purl/1163909 (accessed May 22, 2025).

(27) National Renewable Energy Laboratory (NREL). Biochemical Sugar Model, National Renewable Energy Laboratory (NREL), Golden, CO (United States), 2017, https://www.nrel.gov/extranet/biorefinery/aspen-models/ (accessed Feb 4, 2020).

(28) Humbird, D.; Davis, R.; Tao, L.; Kinchin, C.; Hsu, D.; Aden, A.; Schoen, P.; Lukas, J.; Olthof, B.; Worley, M.; Sexton, D. Process Design and Economics for Biochemical Conversion of Lignocellulosic Biomass to Ethanol: Dilute-Acid Pretreatment and Enzymatic Hydrolysis of Corn Stover; National Renewable Energy Laboratory (NREL), Golden, CO (United States), 2011, https://www.nrel.gov/docs/fy11osti/47764.pdf (accessed Sep 16, 2022).
